# Van der Waals negative capacitance transistors

**DOI:** 10.1038/s41467-019-10738-4

**Published:** 2019-07-10

**Authors:** Xiaowei Wang, Peng Yu, Zhendong Lei, Chao Zhu, Xun Cao, Fucai Liu, Lu You, Qingsheng Zeng, Ya Deng, Chao Zhu, Jiadong Zhou, Qundong Fu, Junling Wang, Yizhong Huang, Zheng Liu

**Affiliations:** 1 School of Materials Science and Engineering, Nanyang Technological University, Singapore, 639798 Singapore; 2 School of Materials Science and Engineering, Sun Yat-sen University, Guangzhou, 510275 Guangdong China; 3 NUS Graduate School for Integrative Sciences & Engineering, National University of Singapore, Singapore, 117583 Singapore; 4 CINTRA CNRS/NTU/THALES, UMI 3288, Research Techno Plaza, Singapore, 637553 Singapore; 5 Centre for Micro-/Nano-electronics (NOVITAS), School of Electrical and Electronic Engineering, Nanyang Technological University, Singapore, 639798 Singapore; 6 Environmental Chemistry and Materials Centre, Nanyang Environment and Water Research Institute, Singapore, 637141 Singapore

**Keywords:** Electronic devices, Two-dimensional materials, Electronic devices

## Abstract

The Boltzmann distribution of electrons sets a fundamental barrier to lowering energy consumption in metal-oxide-semiconductor field-effect transistors (MOSFETs). Negative capacitance FET (NC-FET), as an emerging FET architecture, is promising to overcome this thermionic limit and build ultra-low-power consuming electronics. Here, we demonstrate steep-slope NC-FETs based on two-dimensional molybdenum disulfide and CuInP_2_S_6_ (CIPS) van der Waals (vdW) heterostructure. The vdW NC-FET provides an average subthreshold swing (SS) less than the Boltzmann’s limit for over seven decades of drain current, with a minimum SS of 28 mV dec^−1^. Negligible hysteresis is achieved in NC-FETs with the thickness of CIPS less than 20 nm. A voltage gain of 24 is measured for vdW NC-FET logic inverter. Flexible vdW NC-FET is further demonstrated with sub-60 mV dec^−1^ switching characteristics under the bending radius down to 3.8 mm. These results demonstrate the great potential of vdW NC-FET for ultra-low-power and flexible applications.

## Abstract (web summary)

The adaptation to atomically thin 2D semiconductors and van der Waals layered ferroelectrics can enable negative capacitance transistors with superior performance and bendability. Here, the authors report flexible negative capacitance transistors based on MoS2 and a ferroelectric dielectric CIPS with a minimum sub-threshold slope of 28 mV/dec and high gain logic invertors.

## Introduction

Boltzmann’s limit is inevitable for traditional metal-oxide-semiconductor field-effect transistors (MOSFETs), which thermodynamically defines the lower limit of the subthreshold swing (SS) of 60 mV dec^−1^ at room temperature^1,2^, and subsequently, sets a barrier to further reduce the power consumption. Therefore, energy efficient device concepts based on scalable materials become the key to meet the great demanding in ultra-low-power applications, such as Internet-of-Things and wearable computing electronics. Negative capacitance (NC) field-effect transistor (NC-FET) has been proposed as one of the promising candidates beyond complementary metal-oxide-semiconductor (CMOS) device that may overcome the thermionic limit of 60 mV dec^−1^ by the internal amplification of gate voltage through ferroelectric materials^3–7^. Owing to their atomically thin body nature, two-dimensional (2D) transition metal dichalcogenides (TMDs) have been demonstrated to provide superior immunity to short-channel-effects^8–11^ and suggested to achieve steep subthreshold slope over a wide voltage range for the NC-FET^12–14^. NC-FETs have been reported with TMDs as channel material and ferroelectric hafnium zirconium oxide (HZO)^15–17^ or polymer^14,18^ as ferroelectric gate. Comparing to the bulk ferroelectrics, layered ferroelectrics with atomically smooth surface may offer great performance and high reliability for NC-FETs by minimizing the dangling bonds and charged impurities induced interface traps^19–21^. A few 2D layered materials have been theoretically predicted or experimentally confirmed as ferroelectrics^22–25^. Among them, CuInP_2_S_6_ (CIPS) has been shown with switchable polarization down to 4 nm at room temperature^24^. At the time of writing of this paper, Si et al.^26^ reported the ferroelectric FET based on MoS_2_ and CIPS heterostructure, but the sub-thermionic switching was not demonstrated due to the suspended gate structure.

In this work, we demonstrate the room temperature sub-60 mV dec^−1^ NC-FETs using CIPS flake as the ferroelectric dielectric and atom-thin semiconductor as the channel. The average SS is less than 60 mV dec^−1^ for over seven decades of drain current and the minimum SS is down to 28 mV dec^−1^. The hysteresis window in vdW NC-FET is suppressed by decreasing the thickness of CIPS or incorporating a thin hexagonal boron nitride (h-BN) layer into the NC gate stack. High-gain logic inverter based on vdW NC-FET is built. Bending tests show that sub-60 mV dec^−1^ SS can be retained and hysteresis alleviated for vdW NC-FET on polyester substrate under a bending radius down to 3.8 mm, benefiting from the intrinsic high flexibility and stretchability of 2D materials.

## Results

### Device design and heterostructure characterization

**Fig. 1:**
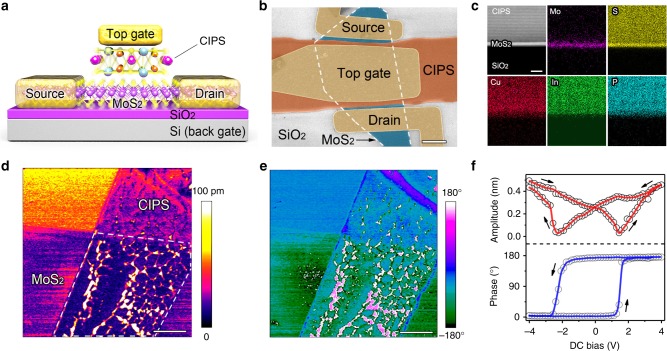
CIPS/MoS_2_ vdW heterostructure and NC-FET. **a**, **b** Schematic diagram (**a**) and False-color SEM image (**b**) of a CIPS/MoS_2_ vdW NC-FET. Scale bar, 2 μm. **c** Cross-sectional high-resolution TEM image of a vertically stacked CIPS/MoS_2_ heterostructure on SiO_2_/Si substrate and corresponding EDS elemental map showing the distribution of Mo, S, Cu, In, and P. Scale bar, 5 nm. **d**, **e** PFM amplitude (**d**) and phase (**e**) of a CIPS/MoS_2_ vdW heterostructure. The CIPS/MoS_2_ stacked region is enclosed by dashed lines in (**d**). Scale bar, 2 μm. **f** The off-field PFM amplitude (top) and phase (bottom) hysteresis loops during the switching process for CIPS flake

The schematic structure of a CIPS/MoS_2_ vdW NC-FET is shown in Fig. 1a, consisting of a few-layer MoS_2_ as the channel material, CIPS flake and 285 nm-thick SiO_2_ as the top NC and back MOS gate dielectric, respectively, heavily doped silicon substrate as the MOS gate electrode and Cr/Au as the NC gate electrode and source/drain contacts (the detailed gate-stack structure of a typical CIPS/MoS_2_ vdW NC-FET is provided in Supplementary Fig. 1). The top-view layout of the devices is given in the false-color scanning electron microscopy (SEM) image (Fig. 1b), where the channel length is slightly larger than the top gate length.

The cross-sectional transmission electron microscope (TEM) image in Fig. 1c shows the layered structure of a typical vdW ferroelectric/semiconductor heterostructure created with atomically flat CIPS and MoS_2_ flakes via the dry transfer process^27^ (Supplementary Note 1 and Supplementary Fig. 2). An atomically sharp and chemically clean interface is achieved between the vdW ferroelectric and semiconductor. The high interface quality would enable the vdW NC-FET with good performance since the NC effect is strongly correlated to interface ferroelectric domain switching. Energy-dispersive X-ray spectrometry (EDS) elemental map presented in Fig. 1c confirms the uniform distribution of Mo, S, Cu, In, and P. The ferroelectricity of CIPS was investigated using piezoresponse force microscopy (PFM) under dual AC resonance tracking (DART) mode (details about the DART mode PFM are provide in Supplementary Note 2 and Supplementary Fig. 3 and 4). The bright and dark regions arising from upward and downward polarizations of CIPS are clearly observed in both amplitude (Fig. 1d) and phase (Fig. 1e) images of local piezoresponse. The off-field PFM amplitude and phase hysteresis loops at individual point during the switching process are presented in Fig. 1f (see Supplementary Fig. 5 for the raw data). The butterfly loop in PFM amplitude and 180° phase change in the nearly square PFM phase loop confirm the good ferroelectric switching nature of CIPS. Single frequency PFM (Supplementary Fig. 6), polarization switching (Supplementary Fig. 7) and polarization versus voltage (*P*-*V*) hysteresis loop measurements (Supplementary Fig. 8) were also conducted to demonstrate the room temperature ferroelectricity in CIPS. Layer number of MoS_2_ was determined by Raman spectroscopy (Supplementary Figs. 9 and 10).

### Electrical measurement of vdW NC-FETs

**Fig. 2:**
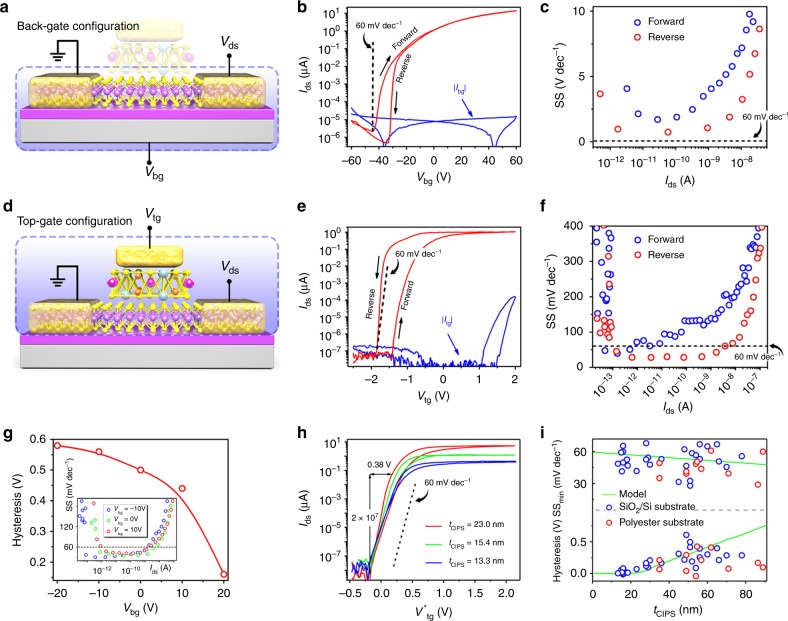
Room temperature electric characterization of CIPS/MoS_2_ vdW NC-FETs. **a** Schematics of the characterization configuration for back-gate measurements. **b**, **c** Back-gate *I*_ds_−*V*_bg_ characteristics (red) and leakage current (blue) (**b**) and SS−*I*_ds_ characteristics (**c**) of a CIPS/MoS_2_ NC-FET. *V*_ds_ = 0.5 V. **d** Schematics of the characterization configuration for top-gate measurements. **e**, **f** Top-gate *I*_ds_−*V*_tg_ characteristics (red) and leakage current (blue) (**e**) and SS−*I*_ds_ characteristics (**f**) of the same device as in (**b**). **g** Ferroelectric hysteresis dependence on *V*_bg_. Inset: SS extracted from the top-gate *I*_ds_−*V*_tg_ characteristics at various *V*_bg_. **h** Top-gate transfer characteristics of vdW NC-FETs with different thickness of CIPS. V^*^_tg_ = *V*_tg_-*V*_th_, where *V*_th_ is the threshold voltage measured with top gate. **i** CIPS thickness dependence of SS (top) and hysteresis width (bottom). Symbol, experimental data; Line, simulation

The room temperature electrical performance of a four-layer MoS_2_ device with the CIPS thickness of 51 nm, channel length of 5.7 μm and width of 5.1 μm and top gate length 3.2 μm is shown in Fig. 2a–f. Figure 2a shows the schematic of back-gate measurement configuration with 285 nm SiO_2_ as the gate dielectric and top gate floating. The *I*_ds_−*V*_bg_ characteristics in Fig. 2b show a typical n-type behavior with an on/off ratio of 10^7^. The clockwise hysteresis between the forward and reverse sweeps can be attributed mainly to charge trapping at the interface of SiO_2_/MoS_2_ and MOS_2_/adsorbates^28^ and is suppressed to half of the original value through vacuum annealing (Supplementary Fig. 11). As shown in Fig. 2c, the minimum SS of MoS_2_ MOSFET is derived to be 1.698 V dec^−1^ for forward sweep and 0.731 V dec^−1^ for reverse sweep according to SS = ∂*V*_bg_/(log*I*_ds_). Both values are far above the thermionic limit at room temperature due to the poor gate efficiency. Contrastingly, for top-gate measurement with CIPS as the ferroelectric gate insulator, so called NC-FET as illustrated in Fig. 2d, the *I*_ds_−*V*_tg_ characteristics (linear scale plot of the *I*_ds_−*V*_tg_ curve is provided in Supplementary Fig. 12) exhibit a sustained sub-60 mV dec^−1^ switching via the internal gate voltage amplification in NC capacitor. The conversion from clockwise hysteresis loop (Fig. 2b) to anticlockwise one (Fig. 2e) by top gating is a result of ferroelectric nature of CIPS and the hysteresis is found to be suppressed by reducing the *V*_tg_ sweep speed (Supplementary Fig. 13). Compared with SiO_2_ gating, the off-state current is significantly reduced in NC-FET due to the trap-free vdW interface between MoS_2_ and CIPS, and the same on/off ratio is achieved despite the limited on-state current by ungated channel segments. SS extracted from the transfer characteristics of NC-FET falls below the thermionic limit for both forward and reverse sweeps, with a minimum of 39 and 28 mV dec^−1^, respectively. Incompletely compensated upward polarization in CIPS due to the low hole concentration in MoS_2_ leads to a larger SS for forward sweep^18^. The average SS for reverse sweep is <60 mV dec^−1^ for over five decades of drain current. The effectiveness of NC effect in vdW NC-FETs is also supported by the observed drain-induced-barrier-rising effect and negative-differential-resistance characteristics (Supplementary Note 3 and Supplementary Fig. 14), which are distinctive features not seen in the conventional MOSFETs. P-type vdW NC-FETs with sub-60 mV dec^−1^ SS were also demonstrated with electrically doped WSe_2_ as the channel material (Supplementary Fig. 15).

We then examine the impact of back-gate biasing on the top-gate transfer characteristics of NC-FET. We found that the ferroelectric hysteresis can be suppressed by positive *V*_bg_ (Fig. 2g) while the SS is slightly improved by negative *V*_bg_ (inset in Fig. 2g). We ascribe these effects to the back-gate modulation of CIPS capacitance (*C*_CIPS_). A vdW NC-FET can be represented as an underlying 2D FET in series with a ferroelectric CIPS capacitor. Therefore, the internal gate voltage amplification gain is derived as *A*_V_ = |*C*_CIPS_|/(*C*_CIPS_| − *C*_int_), where *C*_int_ is the top-gate capacitance of underlying 2D FET. Then the SS of the vdW NCFET can be expressed as SS_NCFET_ = SS_2DFET_/*A*_V_. To obtain a large *A*_V_ and small SS, *C*_int_ should be very close to |*C*_CIPS_|. However, in order to avoid hysteresis, *C*_int_ must be smaller than |*C*_CIPS_|^29^. The increase of underlying MoS_2_ FET channel charge by applying a positive *V*_bg_ leads to an increase in |*C*_CIPS_|, resulting in a reduced hysteresis and increased SS for NC-FET.

### Device architecture optimization

Optimized vdW NC-FETs were fabricated to achieve steep switching and reduce the hysteresis by controlling the thickness of ferroelectric CIPS layer. Figure 2h shows the transfer characteristics of vdW NC-FETs with 23.0, 15.4, and 13.3 nm CIPS (*I*_ds_–*V*_tg_ characteristics of NC-FETs with 29.0 and 20.0 nm CIPS are provided in Supplementary Fig. 16). A minimum SS of 41.8 mV dec^−1^ for reverse sweep with a hysteresis of 70 mV at *I*_ds_ = 10 pA is achieved for 23.0 nm CIPS, less than the one of 453 mV for 51.0 nm CIPS shown in Fig. 2e. The average SS during reverse sweep is less than 60 mV dec^−1^ for over seven decades of drain current, which is three orders of magnitude greater than that of TMD NC-FETs with bulk ferroelectric^15–17^ (Supplementary Table 1). The great transistor performance can be attributed to the strong NC effect due to the trap-free CIPS/MoS_2_ interface in vdW NC-FET. As the thickness of CIPS decreases, its drain current range for SS <60 mV dec^−1^ (over five decades for 15.4 nm CIPS and less than one decade for 13.3 nm CIPS) deteriorates. Nevertheless, the hysteresis of vdW NC-FET with a 13.3 nm CIPS is suppressed to a negligible value (3.4 mV) while the minimum SS (20.6 mV dec^−1^ for forward sweep and 48.6 mV dec^−1^ for reverse sweep) are still less than the thermionic limit. The effect of CIPS thickness on hysteresis and SS can be explained by the size effects on ferroelectricity of CIPS^30^ and capacitance matching between C_CIPS_ and C_int_. Thinning CIPS leads to a decrease in the remnant ferroelectric polarization, the steepness and width of the hysteresis loops, as shown in Supplementary Fig. 17, resulting in a larger SS and smaller hysteresis. Moreover, |*C*_CIPS_| increases with decreasing the thickness of CIPS, leading to a small gate-voltage amplification *A*_V_ and approaching to the hysteresis-free condition for NC-FET (|*C*_CIPS_| > *C*_int_). More than 28 vdW NC-FETs on SiO_2_/Si substrate have been successfully fabricated with CIPS thickness from 13 to 80 nm, and CIPS thickness dependence of SS and hysteresis are summarized in Fig. 2i. Most devices (21 devices) exhibit SS < 60 mV dec^−1^ at room temperature and the main trend of measured SS is captured well by our model simulations (details are provided in Supplementary Note 4 and Supplementary Figure 18–23). According to the simulation results, the design space for sub-60 mV dec^−1^ SS and hysteresis-free operation in a vdW NC-FET gated by a CIPS layer is limited to *t*_CIPS_ < 21 nm, and the SS can only be designed to 57 mV dec^−1^ to avoid hysteresis.

**Fig. 3:**
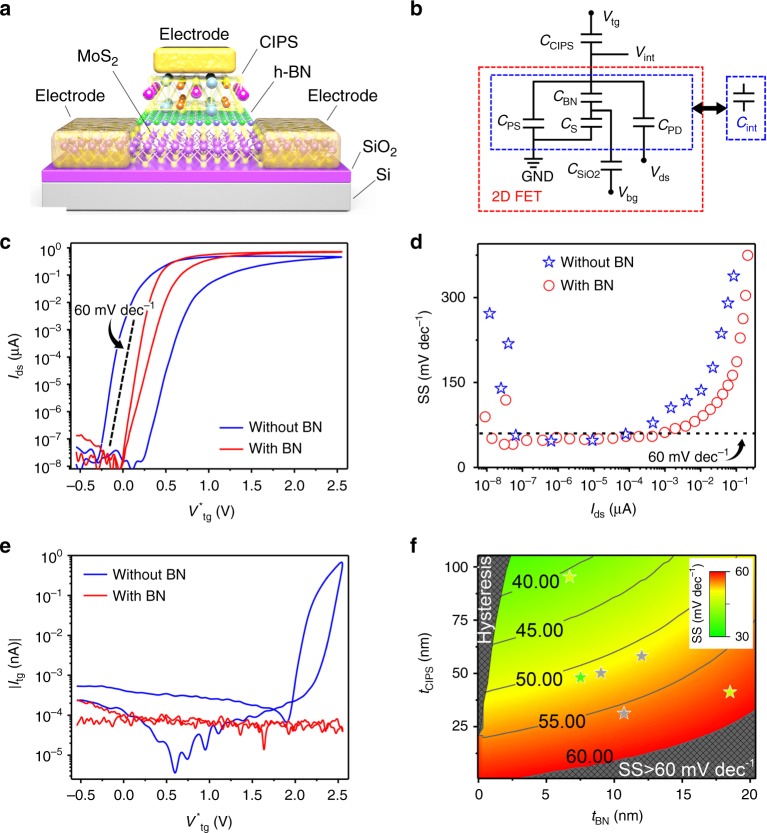
Electric characterization of CIPS/BN/MoS_2_ vdW NC-FETs. **a**, **b** Schematic diagram (**a**) and equivalent capacitor network (**b**) of a CIPS/BN/MoS_2_ vdW NC-FET. **c** Top-gate transfer characteristics of vdW NC-FETs with and without interfacial h-BN layer. V^*^_tg_ = *V*_tg_−*V*_th_. Thickness of CIPS in CIPS/MoS_2_ NC-FET is 49 nm and in CIPS/BN/MoS_2_ NC-FET is 48 nm. The thickness of BN layer is 7.5 nm. **d**, **e** Top-gate SS−*I*_ds_ characteristics for reverse sweep (**d**) and leakage current (**e**) of CIPS/MoS_2_ and CIPS/BN/MoS_2_ vdW NC-FETs. **f** Contour plot of simulated SS as a function of thickness of CIPS (*t*_CIPS_) and BN layer (*t*_BN_) at *V*_ds_ = 0.5 V and *V*_bg_ = 0 V. Symbol, experimental data

In order to further optimize the performance of NC-FET, thin h-BN layers were integrated to the top-gate stack for capacitance matching and gate leakage current reduction, as illustrated in Fig. 3a. From the simplified equivalent capacitance network in Fig. 3b, incorporating a 7.5 nm BN layer (see Supplementary Fig. 24 for atomic force microscopy (AFM) characterization) into the top-gate stack can improve the capacitance matching between CIPS and underlying 2D FET by reducing *C*_int_, and thus leading to a suppression of hysteresis from 607 to 98 mV and negligible degradation of SS for reverse sweep, as shown in Fig. 3c, d, respectively. At the same time, the gate leakage current is reduced by more than 3 orders as shown in Fig. 3e. The design space for vdW NC-FET with BN interfacial layer was also explored using our compact model. The color area represents the design space for vdW NC-FET with non-hysteresis and sub-60 mV dec^−1^ SS. Obviously, the design space is considerably enlarged by integrating the thin BN into the gate stack. The simulation results show a hysteresis-free characteristic for vdW NC-FET with 7.5 nm BN and 48 nm CIPS, while a hysteresis of 98 mV is observed in Fig. 3c. The deviation between experimental and model results can be explained by the non-uniformity in potential and charge at the CIPS/BN interface due to the absence of interfacial metal layer in the real device^31^.

### VdW NC-FET inverters

**Fig. 4:**
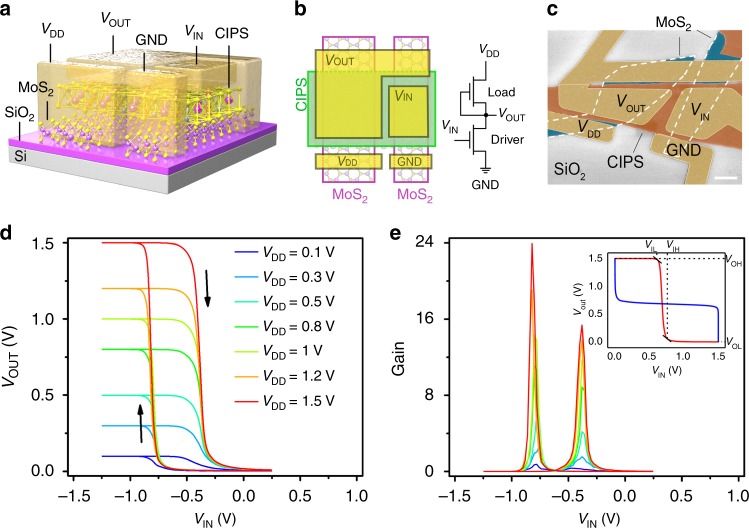
Electrical performance of vdW NC-FET inverter. **a–c** Schematic structure (**a**), circuit schematic (**b**) and false-color SEM image (**c**) of a vdW NC-FET inverter. *W*/*L* = 5.4/4.0 for load NC-FET and *W*/*L* = 2.4/5.5 for driver NC-FET. The thickness of CIPS flake is 42 nm. Scale bar, 2 μm. **d** Room temperature voltage transfer characteristics, *V*_OUT_−*V*_IN_, of the logic inverter measured at various *V*_DD_. **e** Voltage gain of the inverter at various *V*_DD_. Inset: Noise margins of vdW NC-FET inverter at *V*_DD_ = 1.5 V

A logic inverter was fabricated to evaluate the feasibility of vdW NC-FET for low-power applications. As shown in the schematic (Fig. 4a, b), the logic inverter was constructed with two CIPS/MoS_2_ vdW NC-FETs connected in series, serving as the pull-up load and pull-down driver, respectively. The pull-up load was realized by directly connecting the top gate of a NC-FET to the common source electrode. A typical vdW NC-FET inverter with *W*/*L* = 5.4/4.0 for load NC-FET and *W*/*L* = 2.4/5.5 for driver NC-FET, is shown in the false-color SEM image of Fig. 4c, where *W* and *L* denote the width and length of the transistor channels, respectively. Figure 4d presents the voltage transfer curves, plot of input (*V*_IN_) versus output voltage (*V*_OUT_), of vdW NC-FET inverter under various supply voltages (*V*_DD_). Signal inversions are clearly observed with high *V*_OUT_ at low *V*_IN_ even though the *V*_DD_ is down to 0.1 V for both forward and reverse sweeps. Comparing the *V*_OUT_ versus *V*_IN_, a maximum voltage gain as ∼24 can be obtained for *V*_DD_ = 1.5 V, which is considerably higher in comparison with TMDs based MOS inverters^32–34^. The noise margins of the inverter, NM_L_ = 0.406*V*_DD_ and NM_H_ = 0.493*V*_DD_ ((see Supplementary Note 5 and Supplementary Fig. 25 for determination of the noise margins), approach the idea noise margin (0.5*V*_DD_), indicating that the vdW NC-FET inverter is highly immune to electrical noise from the environment and very desirable for integration into multi-stage logic circuits, despite a hysteresis of 380 mV induced by the poor capacitance matching and the intrinsic negative-differential-resistance effect in NC-FET.

### VdW NC-FETs on flexible substrate

**Fig. 5:**
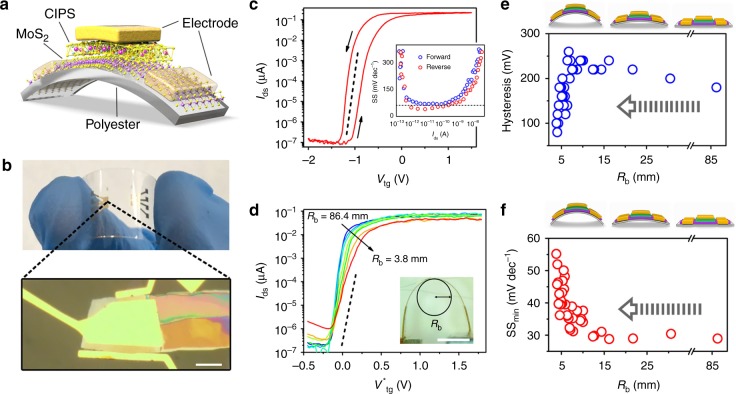
VdW NC-FETs on flexible substrate. **a**, **b** Schematic structure (**a**) and photograph (**b**) of a CIPS/MoS_2_ vdW NC-FET on a flexible polyester substrate. The thickness of CIPS flake is 86.4 nm. Scale bar, 10 μm. **c** *I*_ds_−*V*_tg_ characteristics of a flexible vdW NC-FET. *V*_ds_ = 0.5 V. Inset, SS−*I*_ds_ characteristics. **d** Transfer characteristics of a flexible vdW NC-FET measured at the bending states with *R*_b_ values of 86.4, 12.4, 7.5, 5.8, 4.8, 4.2, and 3.8 mm. V^*^_tg_ = *V*_tg_-*V*_th_. Inset, photograph of a vdW NC-FET on stressed polyester substrate. Scale bar, 5 mm. **e**, **f** The effect of bending radius on the hysteresis width (**e**) and SS (**f**) of the vdW NC-FET

A wide range of flexible electronic devices are typically powered by energy harvesting sources, it is necessary to demonstrate the scalability of vdW NC-FETs in flexible electronic applications to minimize the energy consumption. The layered structures of CIPS and TMDs offer a good mechanical flexibility for vdW NC-FET. Figure 5a, b shows the structure and photograph of a flexible MoS_2_ NC-FET with a pure CIPS dielectric layer atop a 130 um thick polyester substrate, respectively. The vdW NC-FET on flexible substrate exhibits a similar performance with an anticlockwise hysteresis loop and sustained sub-60 mV dec^−1^ switching, as shown in Fig. 5c (see Supplementary Figs. 26 and 27 for more vdW NC-FETs on flexible substrate). In order to investigate the stability of the device performance under static tensile strain, electrical characteristics were recorded with flexible NC-FETs under various bending curvature radius. Figure 5d presents the transfer characteristics of a vdW NC-FET measured at tensile bending states with bending radius (*R*_b_) from 86.4 to 3.8 mm (see Supplementary Note 6 and Supplementary Figure 28 for determination of bending radius). The steep switching characteristic with the minimum SS less than 60 mV dec^−1^ was preserved even with *R*_b_ down to 3.8 mm, less than minimum bending radius reported in previous organic ferroelectric devices^35^. The slight decrease in on-state current with decreasing *R*_b_ arises from the tensile strain induced polarization decrease in CIPS, while the increase in off-state current may result from the piezotronic effect of CIPS induced gate leakage current increase^23^, and *I*_tg_–*V*_tg_ characteristics at various bending states are provided in Supplementary Fig. 29. Surprisingly, the hysteresis window of the flexible vdW NC-FET was suppressed to 80 mV as the bending radius decreases to 4 mm, as shown in Fig. 5e, which is most likely due to the reduction of coercive field for stressed CIPS^36^. This conclusion is also supported by the thickness dependence of hysteresis shown in Fig. 2i and Supplementary Fig. 27, where vdW NC-FETs on flexible substrate show relatively smaller hysteresis compared to devices on SiO_2_/Si substrate due to the residual stresses introduced during device fabrication. Figure 5f illustrates the extracted minimum SS of the device as a function of the bending radius. Sub-30 mV dec^−1^ SS can be achieved at the initial states with bending radius larger than 12 mm following a slight degradation of SS as *R*_b_ decreases below 10 mm, which may result from the suppressed ferroelectric polarization due to the electrical breakdown under extreme bending condition^37^. However, it is clearly that all SS_min_ values are better than the thermionic limit until the bending radius reaches 3.8 mm. Bending cycle test was also carried out with another device and sub-60 mV dec^−1^ switching characteristics were maintained up to 500 cycles (Supplementary Note 6 and Supplementary Fig. 30). The successful demonstration of flexible NC-FET with vdW ferroelectric and semiconductor represents a strategy to meet the ultralow-power operation in the emerging wearable computing applications.

## Discussion

In conclusion, NC-FETs have been successfully demonstrated using van der Waals ferroelectrics and TMDs. The adaptation to vdW layered semiconductor and ferroelectric enables the NC-FET with high performance, originating from the clean interface between MoS_2_ and CIPS, and high bendability. The sub-thermionic switching characteristics and the observed drain-induced-barrier-rising effect and negative-differential-resistance characteristics have been confirmed to be the results of NC effect of CIPS. Hysteresis is considerably reduced by integrating a thin h-BN layer to the gate stack and negligible hysteresis is achieved in vdW NC-FETs with the thickness of CIPS less than 20 nm. Moreover, high-gain inverter based on vdW NC-FET is built. Sub-60 mV dec^−1^ SS can be retained and hysteresis alleviated for vdW NC-FETs on flexible substrate under a bending radius down to 3.8 mm. Our work demonstrates that NC-FETs with vdW ferroelectrics and TMDs are a promising architecture for low-power and wearable applications.

## Methods

### VdW NC-FET device fabrication

High-quality single crystals of CIPS, MoS_2_, and WSe_2_ were synthesized by solid state reaction. The 2D flakes were all achieved by mechanical exfoliation from bulk crystals onto heavily doped silicon substrates with a 285 nm SiO_2_ layer. TMD/CIPS and TMD/BN/CIPS heterostructures were produced with a dry transfer technique as reported. Cr/Au (5 nm/80 nm) electrodes were defined using standard e-beam lithography (EBL) process followed by metal thermal evaporation and lift-off process. Ten-nanometers Au film was used as a discharge layer for EBL processes on polyester substrate.

### Characterizations

Layer number of MoS_2_ and WSe_2_ were identified by optical microscopy and micro-Raman spectroscopy (Witec) with a 532 nm laser. Atomic force microscopy (AFM, Bruker Dimension Icon or Asylum Research Cypher S) in a tapping mode was used to characterize the morphology of the CIPS and device. PFM measurements were carried out on a commercial atomic force microscope (Asylum Research Cypher S) under DART mode. Off-field hysteresis loops were obtained by recording the piezoresponse amplitude and phase 1 signals after individual DC bias was turned off. Electrical transport properties of vdW NC-FETs on SiO_2_/Si substrate were measured with an Agilent B1500A Semiconductor Device Parameter Analyzer in a vacuum chamber of 10^−2^ torr. For the bending test, a micro translation stage was used to hold the flexible substrate on both sides and a manual handle to vary the distance between the two ends of the flexible substrate to control the bending curvature. Bending radius was estimated from the optical image of the strained substrate. The electrical properties of flexible NC-FETs at different bending states were recorded with Agilent B1500A in an air environment.

### Simulation

A vdW NC-FET was treated as an underlying 2D FET in series with a ferroelectric capacitor. The transfer characteristics of 2D FET were obtained by solving the Poisson and drift-diffusion equation. Steady-state Landau-Khalatnikov equation was employed to model ferroelectric CIPS capacitor.

## Supplementary information

Supplementary Information

## Data Availability

The data that support the findings of this study are available from the corresponding author upon reasonable request.
