# Supplementary material for: Van der Waals negative capacitance transistors
Source: Nat Commun. 2019 Jul 10;10:3037. doi: 10.1038/s41467-019-10738-4 (PMC6620276; doi:10.1038/s41467-019-10738-4)
Supplement: Supplementary file 1 — Supplementary Information [file 41467_2019_10738_MOESM1_ESM.pdf]

Supplementary Information

Van der Waals negative capacitance transistors

Wang *et al.*

Supplementary Figures

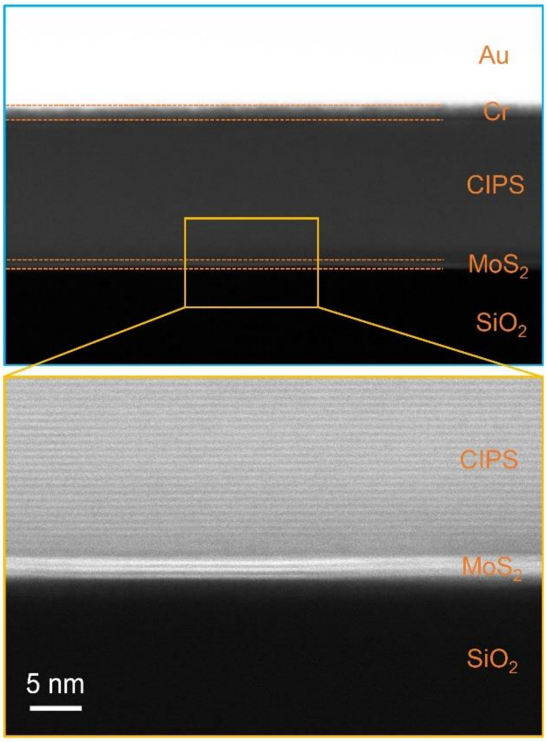

Supplementary Figure 1. Cross-sectional TEM image of a typical CIPS/MoS_2 NC-FET. Scanning TEM image showing the detailed structure (top) and the atomically sharp and chemically clean interface between CIPS and MoS_2 (bottom).

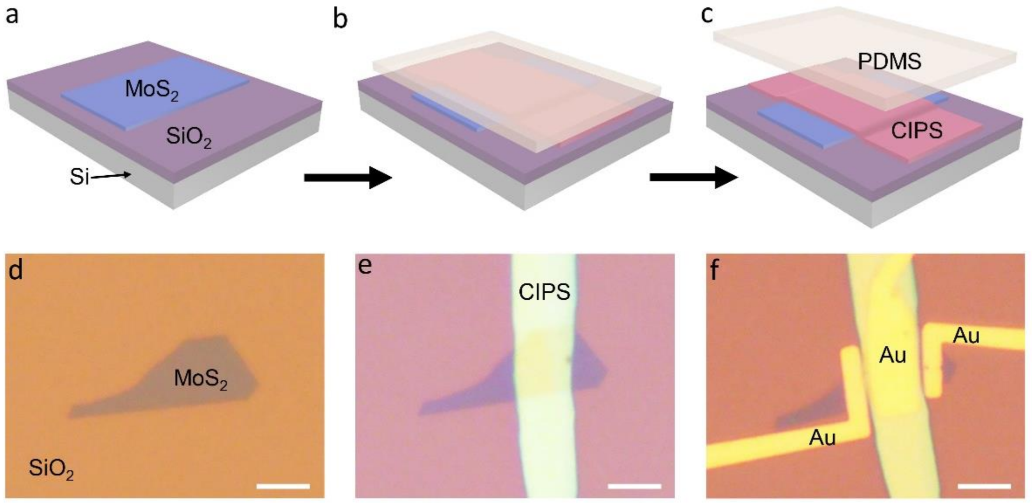

Supplementary Figure 2. Fabrication process of the CIPS/MoS_2 vdW heterostructure based NC-FETs. **a**, **b**, **c**, Schematic fabrication process of CIPS/MoS_2 vdW heterostructure. **d**, **e**, **f**, Optical microscope images of a vdW NC-FET at different process stages. All scale bars are 5 μm.

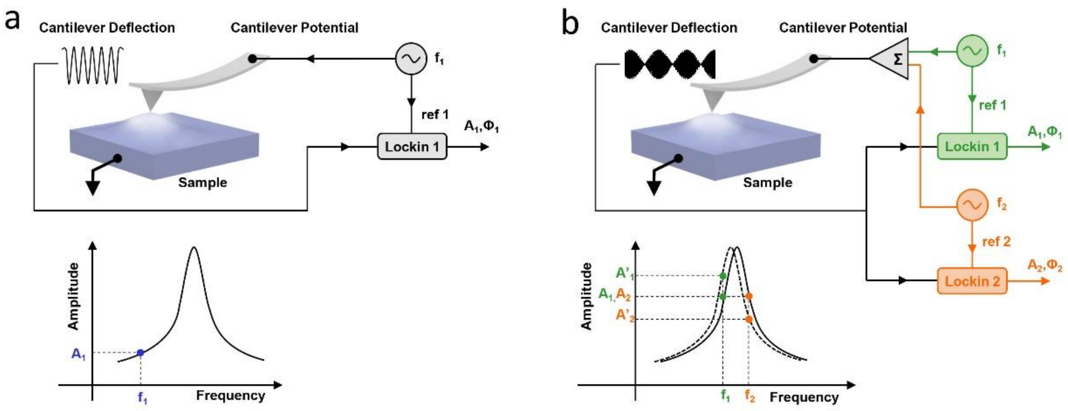

Supplementary Figure 3. Schematic illustration of PFM measurements. a, A diagram of single frequency resonant (SFR) PFM mode. **b**, A diagram of dual AC resonance tracking (DART) mode.

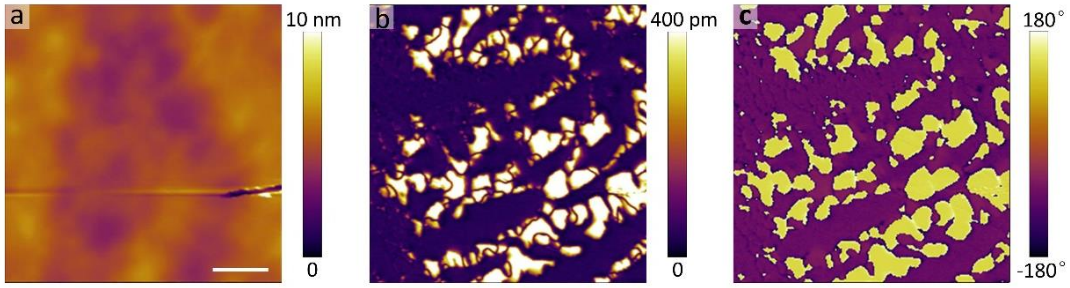

Supplementary Figure 4. DART-PFM images of CIPS. AFM height topography (**a**) DART-PFM amplitude (**b**) and phase (**c**) for a 170 nm CIPS flake. Scale bar, 1 μm.

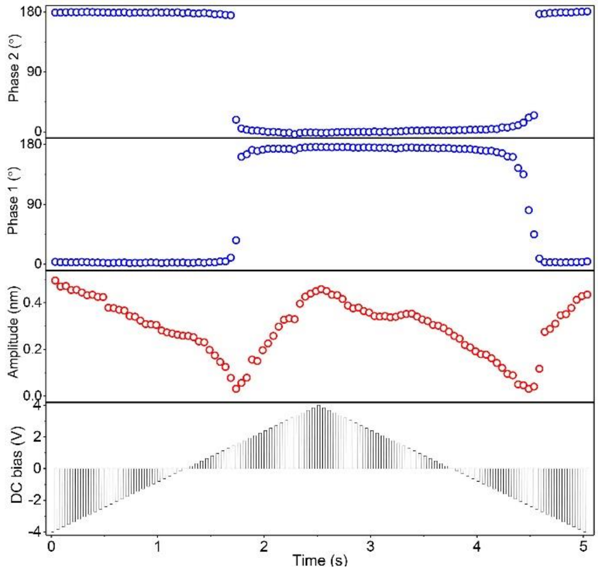

Supplementary Figure 5. Raw data of Figure 1f. DC bias, PFM amplitude, PFM phase 1 and phase 2 of CIPS sample as a function of time.

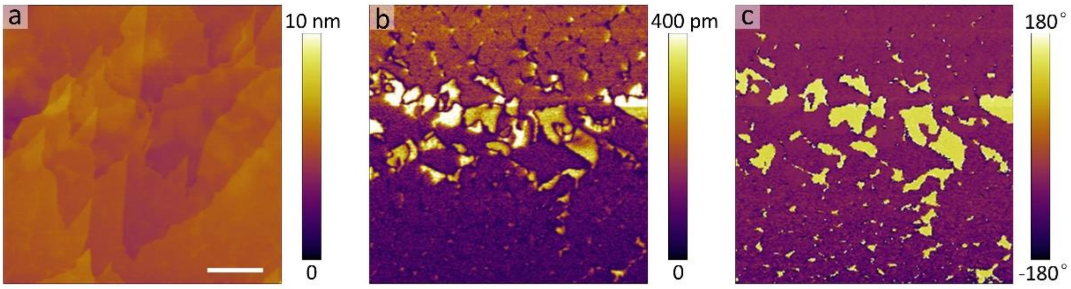

Supplementary Figure 6. SFR-PFM images of CIPS. AFM topography (**a**) SFR-PFM amplitude (**b**) and phase (**c**) of a 124 nm CIPS flake. Scale bar, 1 μm.

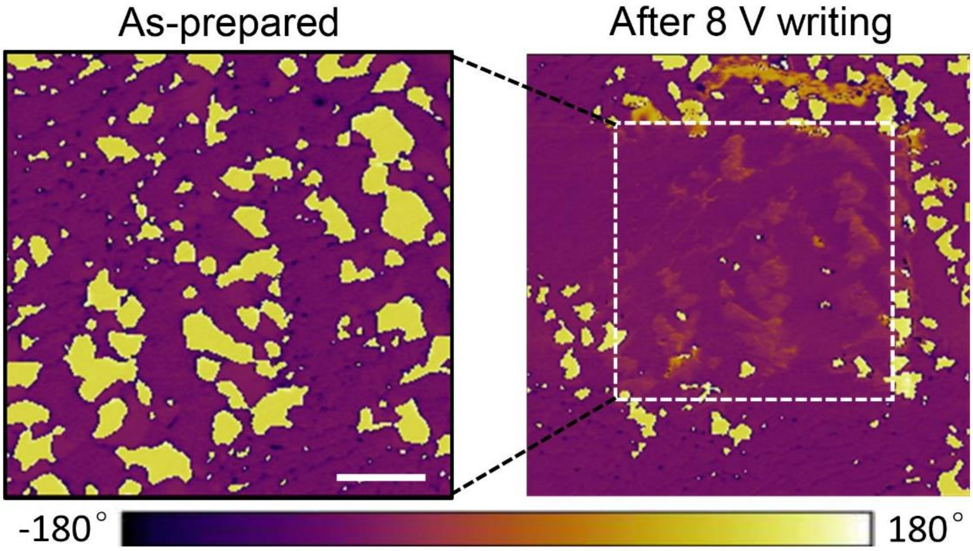

Supplementary Figure 7. Ferroelectric polarization switching in CIPS. The PFM phase images of a 170 nm-thick CIPS flake before (left) and after (right) writing a square pattern at the region enclosed by dashed lines with an 8 V DC bias. Scale bar, 1 μm.

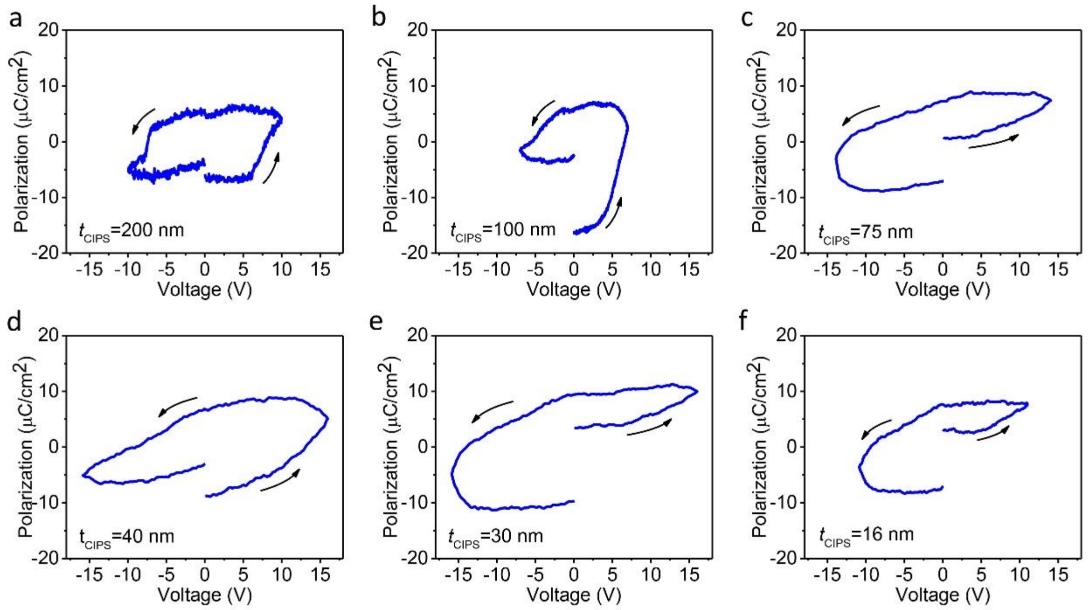

Supplementary Figure 8. *P-V* hysteresis loops of CIPS with different thickness.

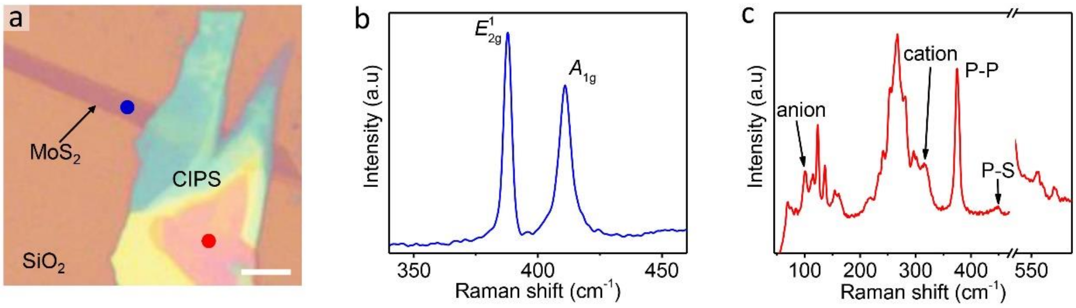

Supplementary Figure 9. Raman spectroscopy characterization of the CIPS/MoS_2 heterostructure. a, Optical image of a CIPS/MoS_2 heterostructure. Scale bar, 5 μm. **b**, **c**, Raman spectra of the different materials as indicated in **a**.

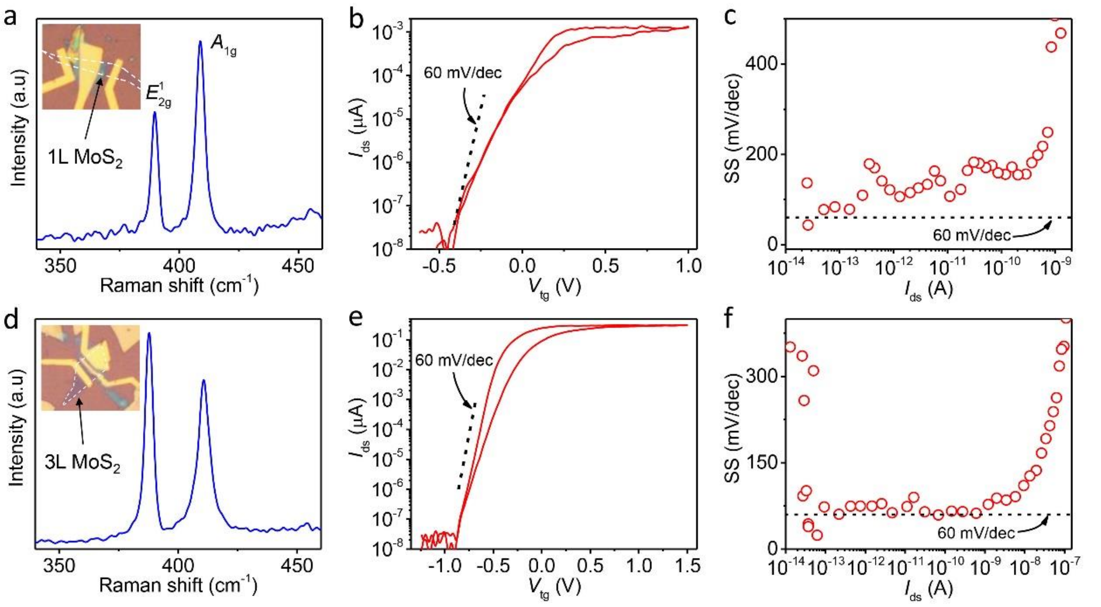

Supplementary Figure 10. Monolayer and trilayer MoS$_2$ NC-FETs. Raman spectrum of monolayer (**a**) and trilayer (**d**) MoS$_2$. Inset: Optical image of the corresponding NC-FET. The I_{ds}–V_{tg} characteristics of monolayer (**b**) and trilayer (**e**) MoS$_2$ NC-FETs. SS versus I$_{ds}$ characteristics of monolayer (**c**) and trilayer (**f**) MoS$_2$ NC-FETs. The thickness of CIPS for monolayer and trilayer NC-FET are 14 nm and 15 nm, respectively.

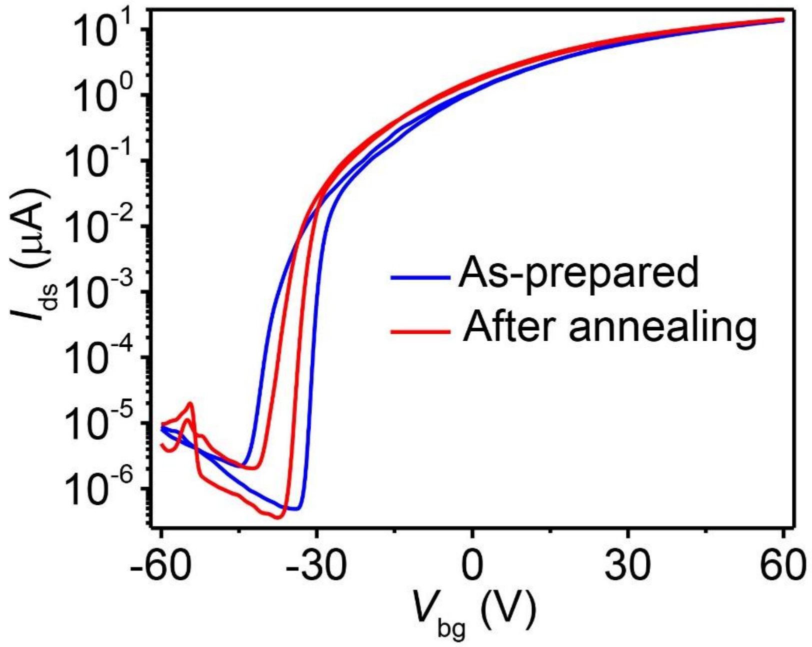

Supplementary Figure 11. Effect of annealing on back-gate hysteresis. Back-gate I_{ds}–V_{bg} characteristics of MoS_2 NC-FET before (blue) and after (red) thermal annealing. Thermal annealing was performed at 200°C for 2 h in a mixture gas of H_2/Ar.

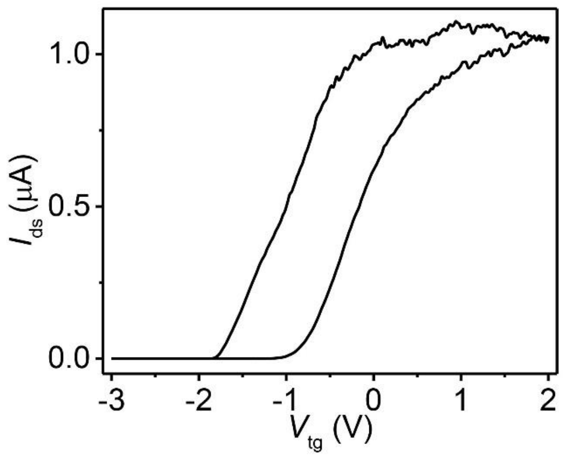

Supplementary Figure 12. Top-gate transfer characteristics in linear scale.

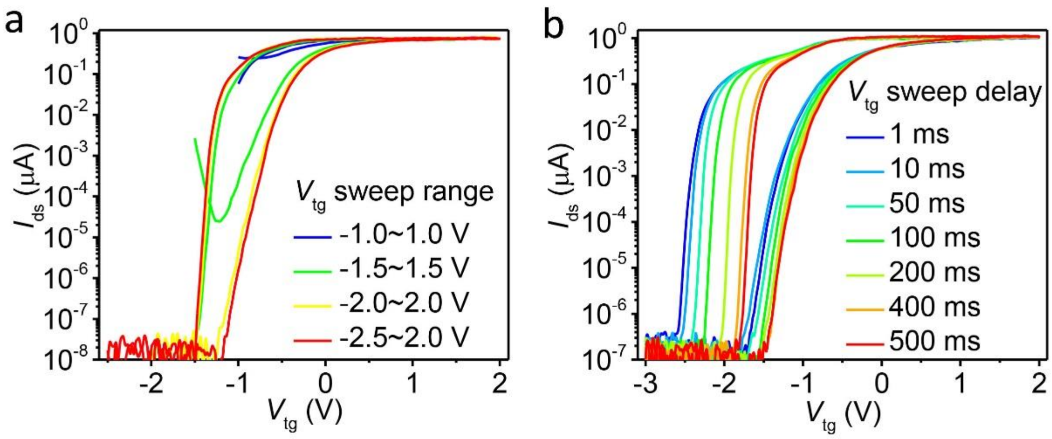

Supplementary Figure 13. Effect of gate voltage sweep ranges and speeds. The transfer curves of the same device as in Figure 3b at different sweep ranges (**a**) and speeds (**b**) of top-gate voltage. V_{ds} = 0.5 V and V_{tg} steps were fixed to be 0.02 V.

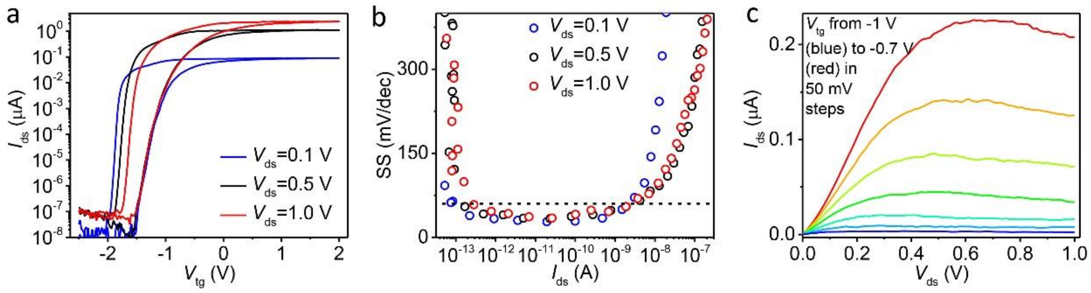

Supplementary Figure 14. DIBR and NDR in vdW NC-FETs. a, The I_{ds}−V_{tg} characteristics of the same device as in Figure 3b at different V_{ds}. **b**, SS versus I_{ds} characteristics extracted from the I_{ds}−V_{bg} curves in **a**. **c**, I_{ds}–V_{ds} characteristics measured from the same NC-FET as in Figure 3b at V_{tg} from −1.0 to −0.7 V in 50 mV steps.

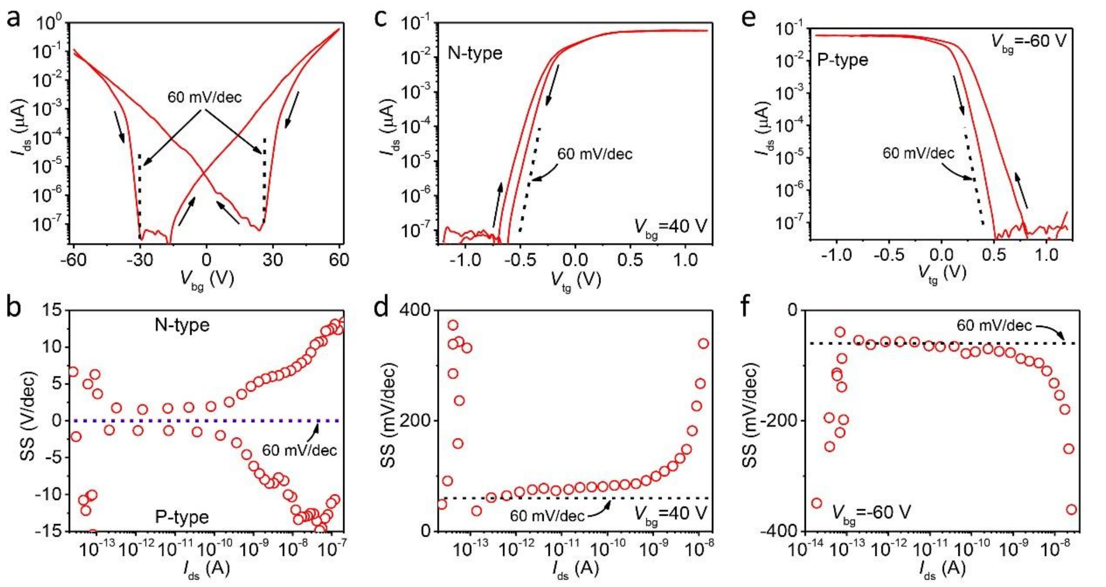

Supplementary Figure 15. WSe_2 vdW NC-FETs. **a**, Back-gate I_{ds}−V_{bg} characteristics of a WSe_2 NC-FET measured with the top gate floating and V_{ds}=0.5 V. The device has a few layer WSe_2 channel with a length L=9.3 μm and width W=6.8 μm. The thickness of the CIPS flake is ~28 nm and the top gate length is 7.3 μm. **b**, SS versus I_{ds} characteristics extracted from the reverse I_{ds}−V_{bg} curve in **a**. **c**, Top-gate I_{ds}−V_{tg} characteristics of the same device as in **a** measured with V_{bg}=40 V and V_{ds}=0.5 V. **d**, SS versus I_{ds} characteristics extracted from the I_{ds}−V_{tg} curve in **c**. **e**, Top-gate I_{ds}−V_{tg} characteristics of the same device as in **a** measured with V_{bg}=-60 V and V_{ds}=0.5 V. **f**, SS versus I_{ds} characteristics extracted from the I_{ds}−V_{tg} curve in **e**.

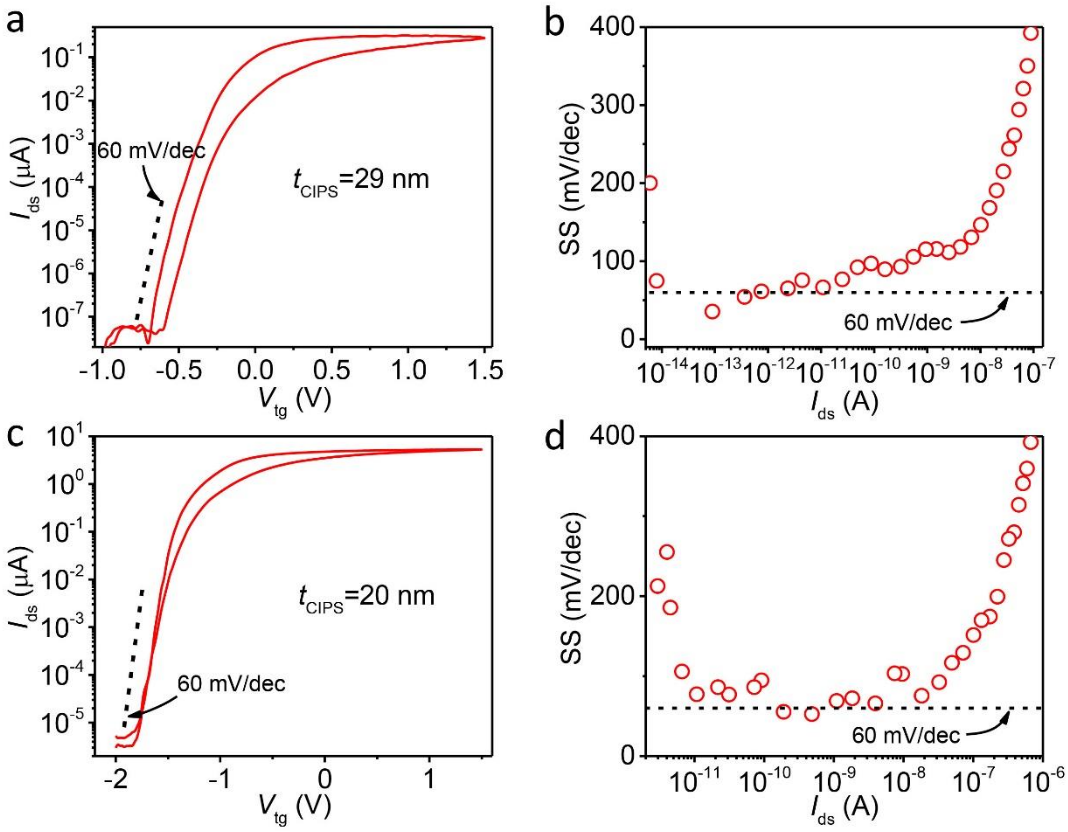

Supplementary Figure 16. CIPS/MoS_2 vdW NC-FETs with different CIPS thickness. I_{ds}–V_{tg} characteristics (**a**) and SS versus I_{ds} characteristics (**b**) of a few layer MoS_2 NC-FET with the CIPS thickness of 29 nm. I_{ds}–V_{tg} characteristics (**c**) and SS versus I_{ds} characteristics (**d**) of a few layer MoS_2 NC-FET with the CIPS thickness of 20 nm.

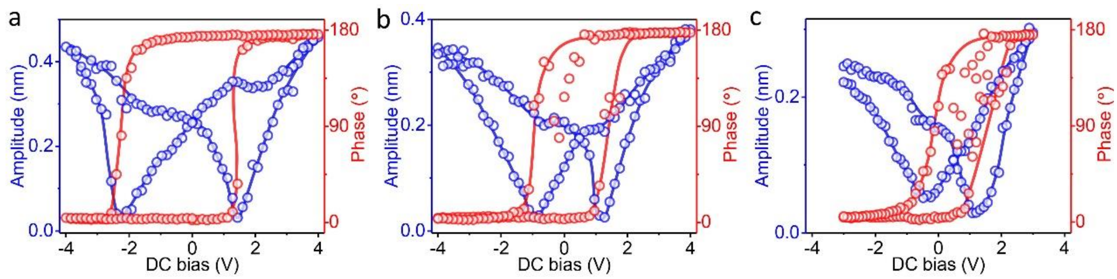

Supplementary Figure 17. Ferroelectric hysteresis loops on CIPS flakes with various thickness. Off-field PFM amplitude (black) and PFM phase 1 (red) hysteresis loops on a (**a**) 51 nm, (**b**) 22 nm and (**c**) 18 nm-thick CIPS flake.

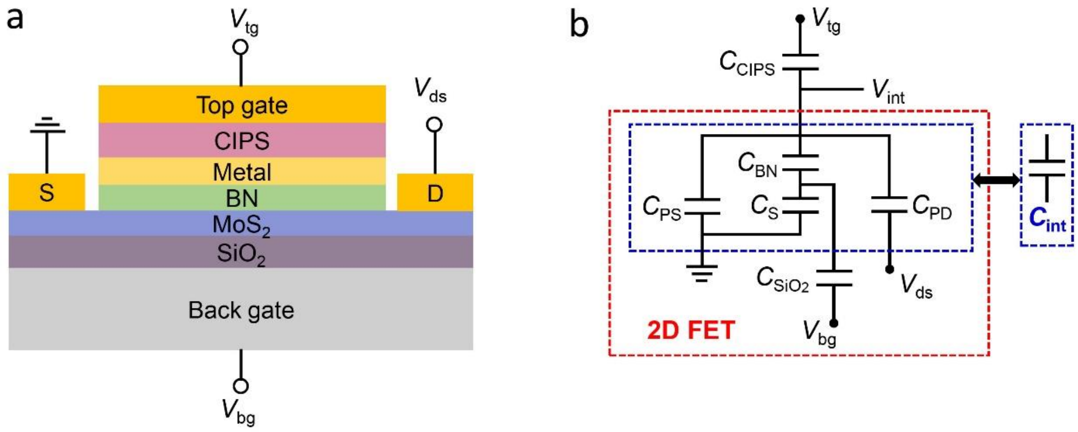

Supplementary Figure 18. Simulation model description. a, Schematic of a vdW NC-FET with BN layer. **b**, Equivalent capacitor network of the NC-FET.

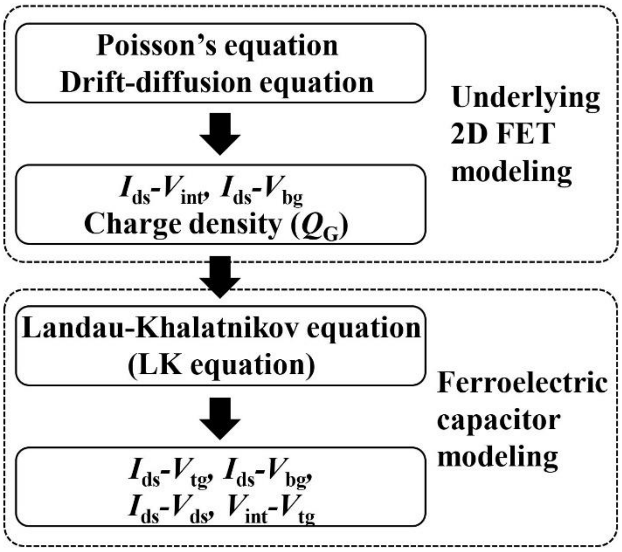

Supplementary Figure 19. Flowchart of the vdW NC-FET simulation.

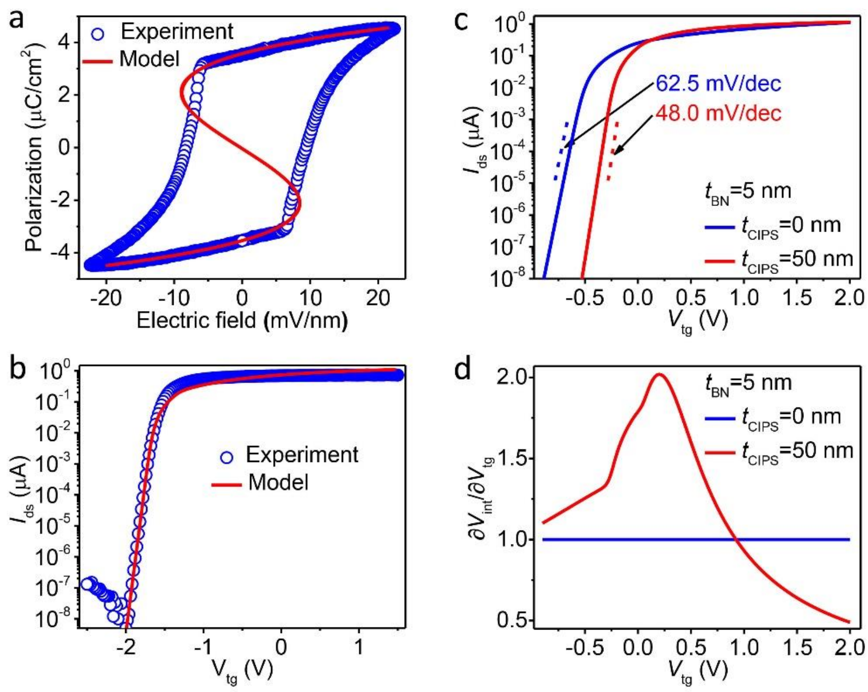

Supplementary Figure 20. Experiments and simulations for a vdW NC-FET and underlying 2D FET. a, Polarization versus electric field (*P*-*E*) curve of a 4 μm-thick CIPS and the fitting curve for Landau coefficients. **b**, Simulated and experimental transfer curves of a vdW NC-FET with 48 nm-thick CIPS and 7.5 nm-thick BN. V_{ds}=0.5 V and V_{bg}=0 V. C_{PS} and C_{PD} are estimated to be 4.72×10^{-3} F/m^2, and 2.36×10^{-3} F/m^2, respectively, according to the small-signal approximation[9]. μ=2 cm^2/Vs. **c**, Simulated transfer curves for a vdW NC-FET (t_{CIPS}=50 nm) and underlying 2D FET (t_{CIPS}=0 nm). **d**, Simulated internal voltage gain as a function of V_{tg} for t_{CIPS}=50 nm and t_{CIPS}=0 nm.

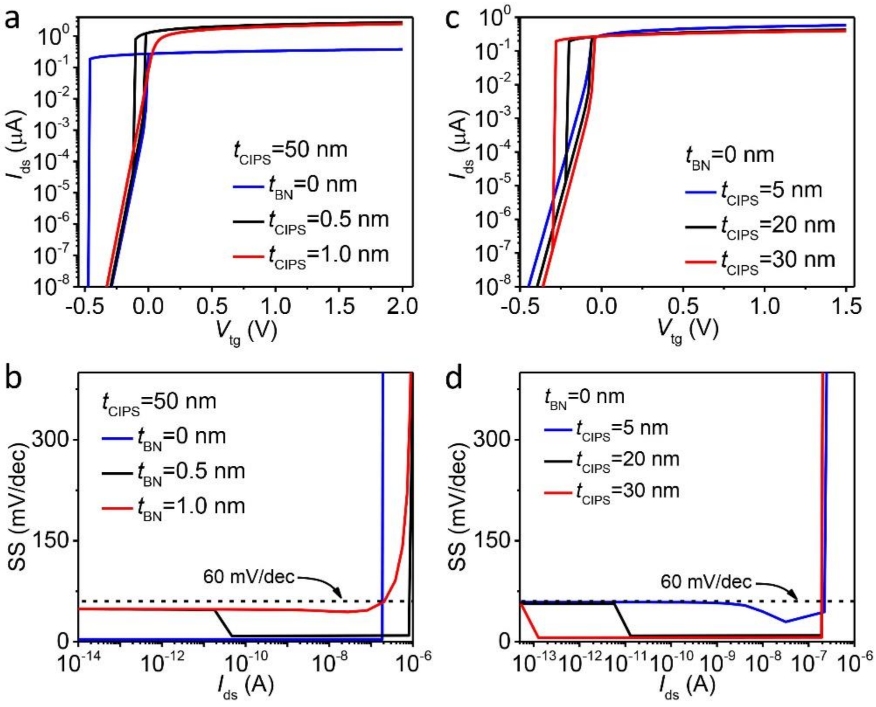

Supplementary Figure 21. Simulated thickness dependence of transfer characteristics. I_{ds}-V_{tg} characteristics (**a**) and SS versus I_{ds} characteristics for reverse sweep (**b**) of vdW NC-FETs with various thickness of BN and 50-nm-thick CIPS. I_{ds}-V_{tg} characteristics (**c**) and SS versus I_{ds} characteristics for reverse sweep (**d**) of vdW NC-FETs without BN layer and with various thickness of CIPS.

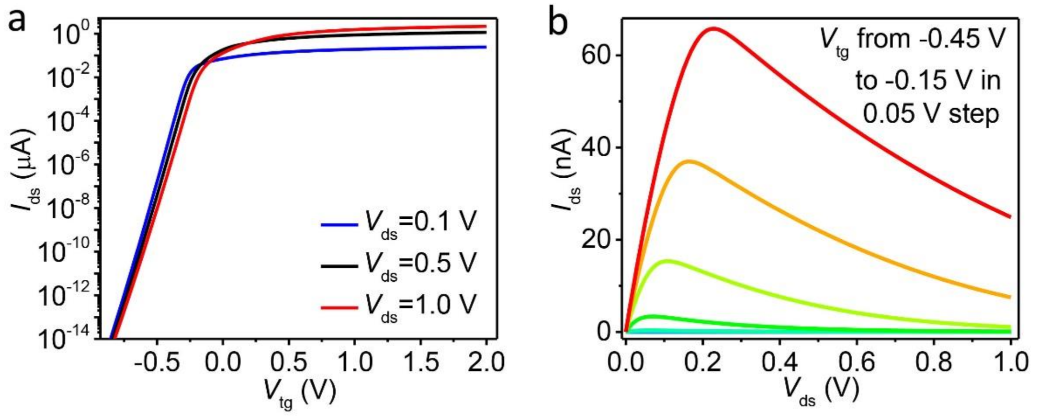

Supplementary Figure 22. Simulation of DIBR and NDR effect in vdW NC-FETs. a, I_{ds}-V_{tg} curves of NC-FET at different V_{ds}. **b**, I_{ds}-V_{ds} curves of NC-FET at different V_{tg}. t_{BN}=5 nm and t_{CIPS}=50 nm.

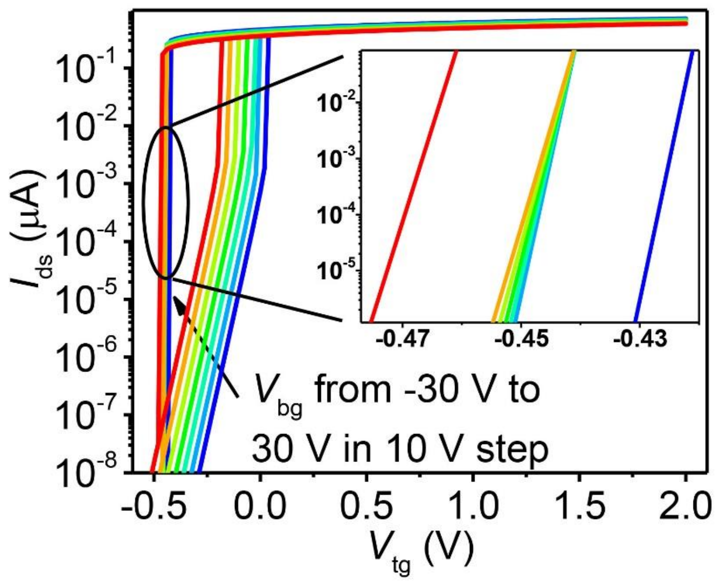

Supplementary Figure 23. Back-gate biasing effects on transfer characteristics of NC-FETs. I_{ds}-V_{tg} characteristics of NC-FET at various V_{bg}. Inset: Zoom-in of the I_{ds}–V_{tg} curve between −0.48 and −0.42 V.

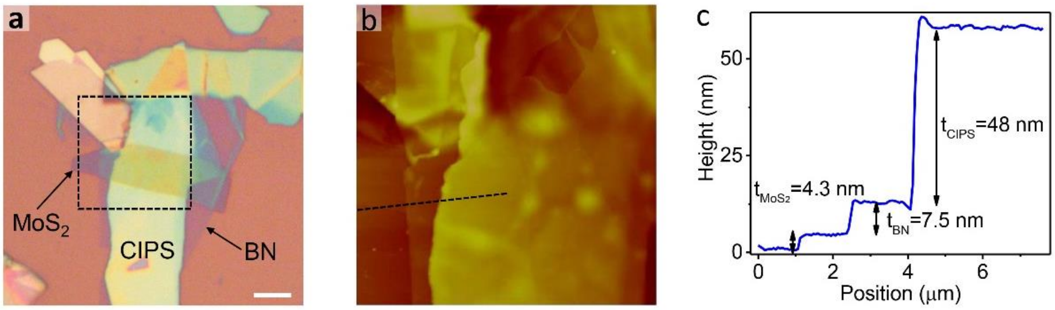

Supplementary Figure 24. AFM characterizations of CIPS/BN/MoS_2 heterostructure. **a**, Optical microscope image of a CIPS/BN/MoS_2 heterostructure, Scale bar, 5 μm. **b**, AFM topography of the region enclosed by dashed lines in **a**. **c**, Height profiles along the dashed line as indicated in **b**.

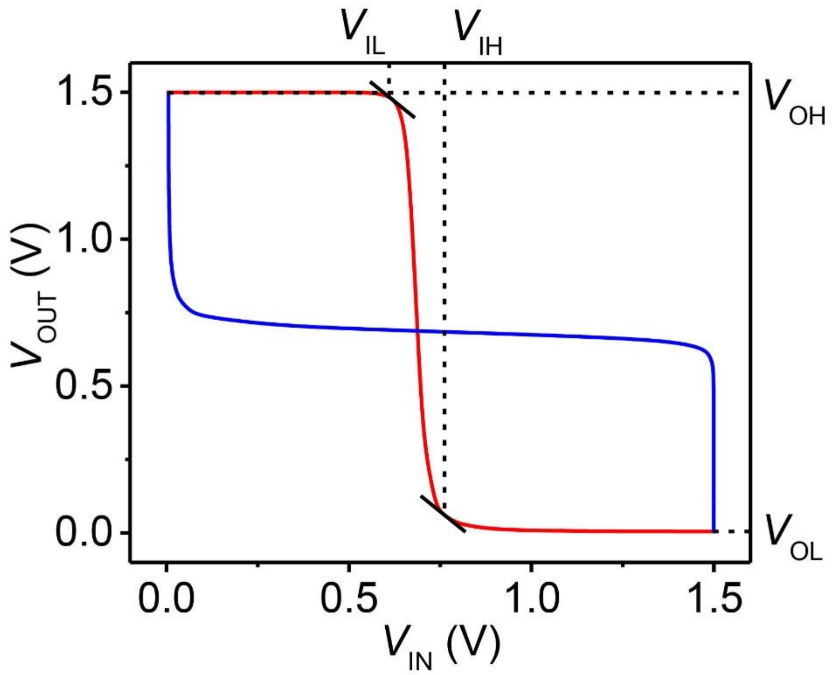

Supplementary Figure 25. Noise margins of vdW NC-FET inverter. Voltage transfer curve and its mirror reflection of a vdW NC-FET inverter shows input low and high (V_{IL} and V_{IH}) and output low and high (V_{OL} and V_{OH}) at V_{DD}=1.5 V.

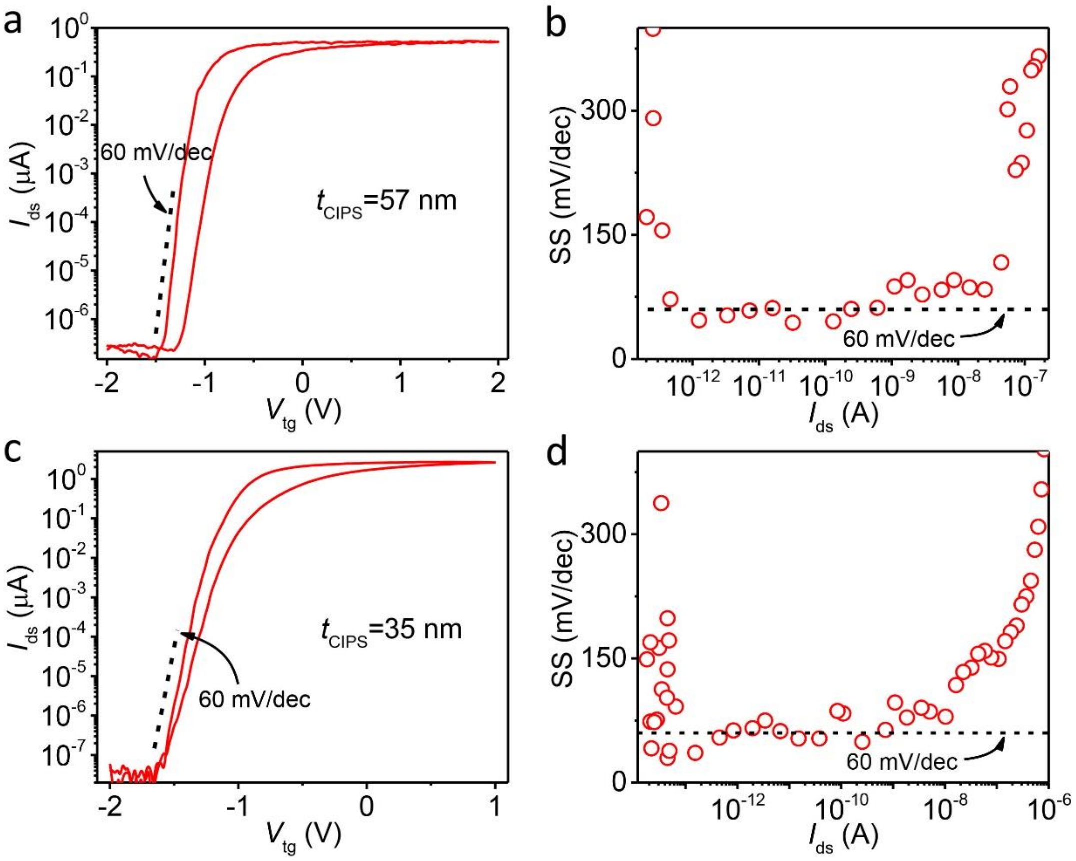

Supplementary Figure 26. VdW NC-FETs on flexible substrate with different CIPS thickness. I_{ds}–V_{tg} characteristics (**a**) and SS versus I_{ds} characteristics (**b**) of a flexible CIPS/MoS_2 NC-FET with the CIPS thickness of 57 nm. I_{ds}–V_{tg} characteristics (**c**) and SS versus I_{ds} characteristics (**d**) of a flexible CIPS/MoS_2 NC-FET with the CIPS thickness of 35 nm.

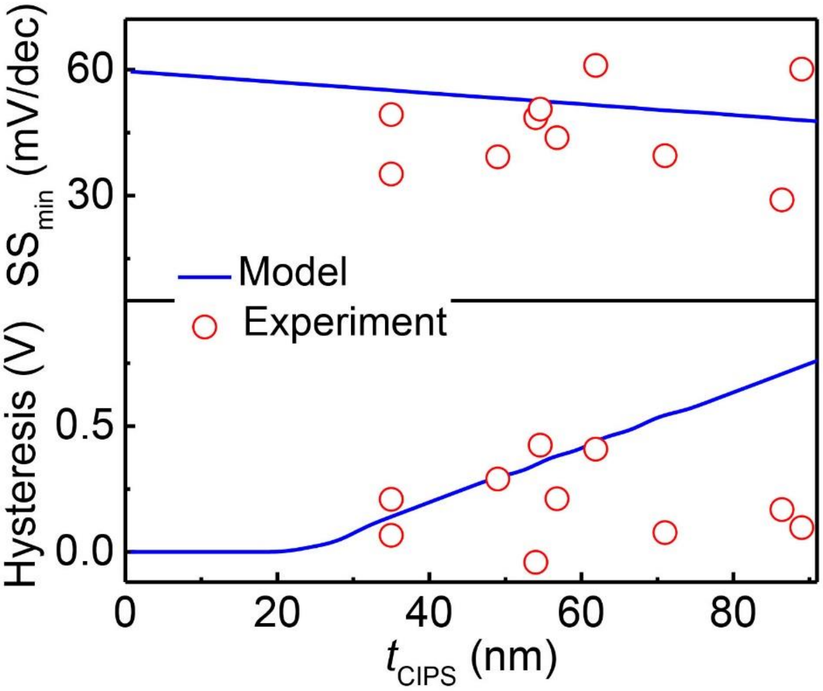

Supplementary Figure 27. Thickness dependence of SS and hysteresis of flexible vdW NC-FETs. CIPS thickness dependence of SS (top) and hysteresis width (bottom). Symbol, experimental data; Line, simulation.

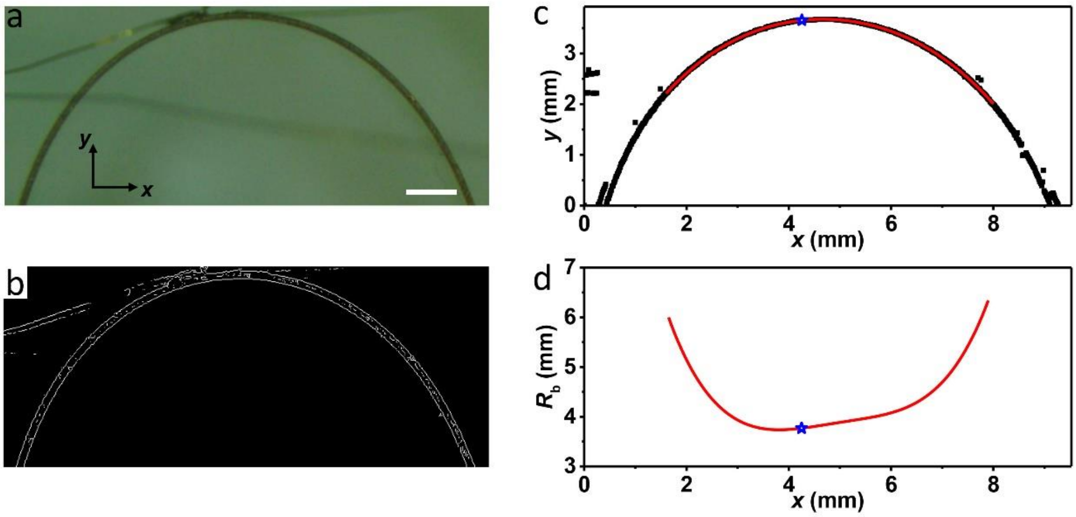

Supplementary Figure 28. Bending radius determination of flexible NC-FET. a, Photograph of a vdW NC-FET on polyester substrate in a tensile bending state. **b**, Image contour extracted from green component of **a**. **c**, Surface height profile (Symbol) of polyester substrate extracted from **b**. Line: Polynomial fitting. **d**, Calculated bending radius profile of polyester substrate. Position of the NC-FET is marked with a blue star in **c** and **d**.

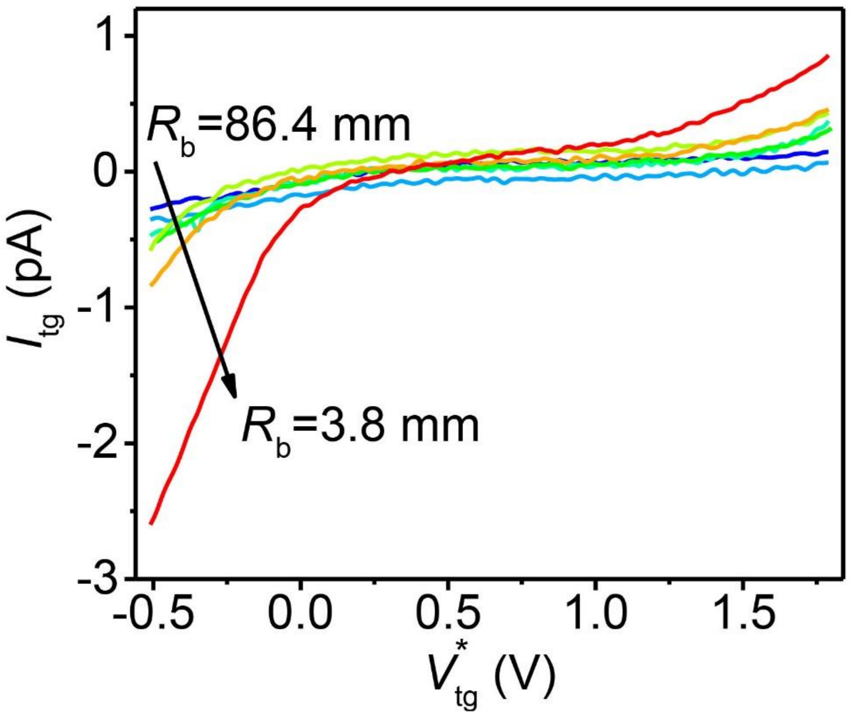

Supplementary Figure 29. Effect of bending radius on top-gate leakage current of flexible NC-FET. Top-gate leakage current of a flexible vdW NC-FET measured at bending states with R_b values of 86.4, 12.4, 7.5, 5.8, 4.8, 4.2 and 3.8 mm.

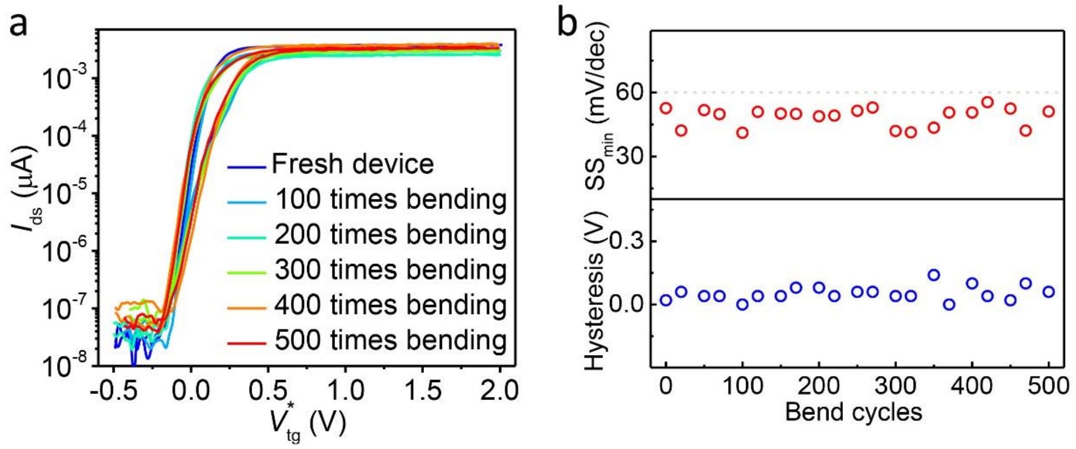

Supplementary Figure 30. Effect of bending cycles on top-gate leakage current of flexible NC-FET. a, Top-gate transfer characteristics of vdW NC-FET measured at flat state after bending five hundred times. The bending radius was manually fixed at ~5 mm and the I_{ds}–V_{tg} curve was measured after the cycles. The thickness of CIPS is 49 nm. **b**, The effect of bending cycles on the SS (top) and hysteresis width (bottom) of the vdW NC-FET.

Supplementary Tables

Steep-slope FET	This work	Ref. 1	Ref. 2	Ref. 3	Ref. 4	Ref. 5
Structure	Top-gate NC-FET	Back-gate NC-FET	Back-gate NC-FET	Top-gate NC-FET	Top-gate NC-FET	DS-FET
Channel	MoS_2, WSe_2	MoS_2	WSe_2	MoS_2	MoS_2	CNT
Ferroelectric	vdW CIPS	$Hf_{0.5}Zr_{0.5}O_2$	$Hf_{0.5}Zr_{0.5}O_2$	P(VDF-TrFE)	P(VDF-TrFE)	None
Thickness of ferroelectric (nm)	13 to 95	20	20	200	50 to 300	None
SS_{min} (mV/dec)	28	$SS_{\#1}$=41.7 $SS_{\#2}$=5.6	14.4	11.7	24.2	35
Current range for SS<60 mV/dec	7	4	3	4	4	4
Hysteresis (mV)	3.4	12	120	Unknown (breakdown after first sweep)	>5000	60
Flexible	Yes (SS<60 mV/dec under a bending radius down to) 3.8 mm	Not	Not	Not	Not	Not

Supplementary Table 1. Comparison with previous studies on steep-slope FETs. The NC-FET with vdW ferroelectric is first time demonstrated to achieve both SS < 60mV/dec and hysteresis free operation. The average SS < 60mV/dec is obtained for over 7 decades of drain current, which is three orders of magnitude greater than that of NC-FETs with bulk ferroelectric.

Supplementary Notes

Supplementary Note 1

VdW heterostructures and NC-FETs fabrication and characterization

Fabrication process of vdW heterostructures and NC-FETs. CIPS/TMD and CIPS/BN/TMD heterostructures were produced with a dry transfer technique as reported previously[6]. Supplementary Figure 2a to c show the schematic of the CIPS/MoS_2 vdW heterostructure fabrication process and Supplementary Figure 2d to f show optical images of a MoS_2 NC-FET at different process stages. A MoS_2 flake is first transferred onto a Si/SiO_2 substrate with a mechanical exfoliation method (Supplementary Figure 2a and d). A CIPS flake was transferred onto a transparent poly-dimethylsiloxane (PDMS) film with the same method and then aligned on the MoS_2 flake with the aid of a micromanipulator under an optical microscope (Supplementary Figure 2b). CIPS was left on MoS_2 flake after PDMS film was lifted off and the CIPS/MoS_2 heterostructure is achieved (Supplementary Figure 2c and e). The transfer process is like stamping a pattern with a stamp. For CIPS/BN/TMD structures, a BN flake was transferred on the TMD before the transfer of CIPS. Finally, Au/Cr electrodes were defined on the obtained heterostructures using e-beam lithography (EBL) (Supplementary Figure 2f). VdW heterostructures on polyester substrate were constructed with the same process as the SiO_2/Si substrate A 10 nm Au film was used as a discharge layer for EBL process on polyester substrate.

VdW heterostructures characterization. Layer number of MoS_2 flakes were

identified by optical microscopy, Raman spectroscopy and AFM measurement. Supplementary Figure 9 shows the Raman spectroscopy characterization of a CIPS/MoS_2 heterostructure. There are two characteristic Raman modes for MoS_2, the in-plane (E^1_{2g}) and the out-of-plane (A_{1g}) vibrational modes with frequency difference $\Delta\omega$ = 23.1 cm^{-1}, indicating a trilayer MoS_2[7], as shown in Supplementary Figure 9b. Raman spectrum of CIPS is shown in Supplementary Figure 9c**.** Peaks in the 90−140, and 300−320 cm^{-1} ranges are assigned to anion ($P_2S_6^{4-}$) and cation (Cu^I, In^{III}) vibrations, respectively. The polar intense peak at 375 cm^{-1} may be ascribed to the P-P stretching mode. P−S oscillations are responsible for peaks in 410−460 cm^{-1} range. The peaks are consistent with the reported Raman spectrum measured in bulk CIPS and the presence of the peak at ~320 cm^{-1} indicates that our CIPS sample is in the ferroelectric phase[8-10].

AFM measurement was also used to check the morphology of the fabricated heterostructures and determine the thickness of TMD, BN and CIPS. Supplementary Figure 24 shows the AFM characterizations of a CIPS/BN/MoS_2 heterostructure. The thickness of MoS_2, BN and CIPS are measured to be ~4.3, 7.5 and 48 nm, respectively.

Supplementary Note 2

Ferroelectricity in $CuInP_2S_6$ flakes

Polarization-voltage (P-V) measurements of CIPS. P-V measurements was also conducted to demonstrate the ferroelectricity in CIPS with different thickness. As can

be seen in Supplementary Figure 8, 200-nm-thick flake still shows square hysteresis loop similar to that of the bulk sample. However, with decreasing thickness, the leakage current starts to become dominant under one of voltage polarities, resulting in the opening-up of the loops. The asymmetric (diode-like) leakage behavior could originate from the polarization-modulated interface band bending. Nevertheless, the fingerprint of the hysteresis loop remains visible down to the lowest thickness.

Piezoresponse force microscopy measurement. To verify the room-temperature ferroelectricity of $CuInP_2S_6$ (CIPS), CIPS flakes with different thicknesses were investigated using piezoresponse force microscopy (PFM) under both single frequency resonant (SFR) and dual AC resonance tracking (DART) modes, as illustrated in Supplementary Figure 3. Supplementary Figure 6 shows the SFR-PFM characterizations of CIPS with a thickness of 124 nm. For SFR-PFM, a frequency close to the contact resonance is selected to drive the cantilever, and the amplitude and phase of the piezo response are measured using a lock-in amplifier, as schemed in Supplementary Figure 3a. A clear contrast arisen from ferroelectric domains is observed in both amplitude and phase of local piezoresponse. 180° phase flip across domain walls in Supplementary Figure 6c confirms out-of-plane polarization orientation in CIPS.

Rather than using the phase as the input to the frequency feedback, the difference between the amplitudes at two drive frequencies is used as the input feedback in DART mode, as schemed in Supplementary Figure 3b, which dramatically reduces topographic crosstalk. Supplementary Figure 4 presents the similar ferroelectric

domain structures in a 170 nm-thick CIPS measured by DART-PFM.

Ferroelectric switching. Polarization switching in CIPS was performed by applying a DC bias between the conductive PFM tip and the heavily doped Si substrate. Clear reversal of phase contrast was observed after writing a square pattern with a positive tip voltage (Supplementary Figure 7), which confirms the switching of polarization in CIPS and rules out the possible electrochemical contribution to PFM signal[11, 12].

PFM hysteresis loops of CIPS flakes with various thickness. The size effect on ferroelectricity was investigated by measuring the off-field PFM amplitude and phase hysteresis loops of CIPS with different thicknesses, shown in Supplementary Figure 17. A thicker CIPS flake show a larger piezoresponse, broader hysteresis loop and steeper phase change, indicating a stronger ferroelectricity. The piezoresponse amplitude, hysteresis loop width and phase change steepness decreases by thinning CIPS, which will result in a degraded SS but alleviated hysteresis for NC-FET with a thinner CIPS dielectric.

Supplementary Note 3

N-type CIPS/MoS$_2$ and p-type CIPS/WSe$_2$ vdW NC-FETs on SiO$_2$/Si substrate

Drain-induced-barrier-rising and negative differential resistance in vdW NC-FETs. Supplementary Figure 14a and b present the I_{ds}–V_{tg} curves of a vdW NC-FET measured at different V_{ds} and the corresponding SS versus I_{ds} characteristics. Threshold voltage for the reverse sweep increases with V_{ds} due to the

drain-induced-barrier-rising (DIBR) effect at the NC region. Meanwhile, hysteresis window is reduced, and SS is increased by applying a larger V_{ds}. An increased drain voltage reduces C_{int} and the gate voltage amplification A_V due to the drain to gate coupling[13, 14], resulting in a lower channel current in the subthreshold region (Supplementary Figure 14a) and larger SS (Supplementary Figure 14b). As discussed in the main text, a vdW NC-FET can be described as an underlying 2D FET in series with a ferroelectric CIPS capacitor. The internal gate voltage amplification gain can be obtained from the simple capacitive divider as $A_V = |C_{CIPS}|/(|C_{CIPS}|-C_{int})$, where C_{int} is the top-gate capacitance of underlying 2D FET. Then the SS of the vdW NCFET is given as $SS_{NCFET}=SS_{2DFET}/A_V$. $|C_{CIPS}|$ and C_{int} should be as close as possible to achieve a large A_V and small SS. In order to avoid hysteresis, C_{int} should be smaller than $|C_{CIPS}|$. So the decrease of C_{int} will improve the capacitance matching between CIPS and underlying 2D FET, leading to a suppression of hysteresis. Because of the DIBR effect, current in the subthreshold region is reduced with increasing V_{ds} and negative differential resistance characteristics is observed in output curves of vdW NC-FET, shown in Supplementary Figure 14c. The observed DIBR effect and NDR characteristics indicate the effectiveness of NC effect in vdW NC-FETs.

Effect of gate voltage sweep ranges and speeds on top-gate transfer characteristics. Because ferroelectric polarization and hysteresis depend on the sweep rate and range, electrical characterizations of vdW NC-FETs at different V_{tg} sweep speeds and ranges were also examined. Supplementary Figure 13a displays I_{ds}–V_{tg} characteristics for the same vdW NC-FET as in Figure 3b of main text measured with

different gate voltage ranges. The hysteresis window becomes larger and the switching slope becomes steeper because of the more complete polarization at a higher gate sweep range. Sweep speed was controlled by modifying the sweep delay from 1 to 500 ms while keeping the voltage step constant. SS of the NC-FET was improved and hysteresis was suppressed by decreasing the sweep speed, as shown in Supplementary Figure 13b.

Current saturation in top-gate transfer characteristics. Supplementary Figure 12 shows the linear scale plot of the Figure 2e of main text. The saturation of drain current at on-state arise from the underlapped top-gate regions near the source/drain electrodes. Drain current I_{ds} can be expressed as $I_{ds} = V_{ds}(G_U/(1 + G_U/G_G))$, where G_U and G_G are the conductance of un-gated and gated MoS_2, respectively. In the top-gate measurement and without back-gate voltage, $G_G \gg G_U$ at on-state and I_{ds} will be limited to $V_{ds}G_U$, which is top gate untunable. This saturation behavior is in contrast to the common velocity saturation and can be eliminated in vdW NC-FET with CIPS as the global back-gate dielectric, where the whole channel is gated through CIPS.

Monolayer MoS_2 vdW NC-FET. The channel materials of all the vdW NC-FETs shown in the main text are few layered MoS_2. NC-FETs with monolayer MoS_2 were also demonstrated and examined, as shown in Supplementary Figure 10a to c. Due to the ungated channel segments and high Schottky barrier at MoS_2/metal contacts, on-state current and SS of the monolayer MoS_2 NC-FET are degraded in comparison

with a trilayer MoS_2 NC-FET with the same thickness of CIPS, as shown in Supplementary Figure 10d to f.

P-type CIPS/WSe_2 vdW NC-FETs. VdW NC-FETs with tungsten diselenide (WSe_2) as channel material were also demonstrated to achieve sub-60 mV/dec p-type switching. Back-gated WSe_2 FET exhibits ambipolar transport characteristics, as shown in Supplementary Figure 15a, because metal Fermi level is close to the middle of the WSe_2 bandgap[15]. The minimum SS for n-type and p-type back-gated WSe_2 FET are 1.53 V/dec and 1.27 V/dec, respectively. N and p-type WSe_2 vdW NC-FETs were obtained by the back-gate electrical doping, as shown in Supplementary Figure 15c and e, with the minimum SS of 37 mV/dec and 40 mV/dec, respectively. The observed anti-hysteresis behaviors (clockwise hysteresis loop) in Supplementary Figure 15c and e can be attributed to the trapping/de-trapping of charge carriers at the SiO_2/Si interface and the polarization screening form the trapped charges[16].

Supplementary Note 4

Simulation of vdW NC-FETs

Simulation approach. Supplementary Figure 18a shows schematic of the CIPS/BN/MoS_2 vdW NC-FET with a MOS back-gate and a NC top gate. The top NC gate is described with a meatal-ferroelectric-metal-insulator-semiconductor structure. The metallic layer between CIPS and BN layers is introduced to average out the non-uniformity in potential and charge at the ferroelectric/insulator interface and act as the internal top gate of underlying 2D FET. Equivalent capacitance model is shown

in Supplementary Figure 18b, with C_{CIPS} the ferroelectric capacitance, C_{int} the top-gate capacitance of underlying 2D FET and $C_{\mathrm{SiO_2}}$ the back-gate capacitance. C_{PS} and C_{PD} represent the parasitic capacitance of internal gate to source and drain[14], respectively. The internal voltage (V_{int}) indicates the voltage amplified by the ferroelectric CIPS, and it acts as the top-gate voltage of the underlying FET. The overall simulation procedure shown in Supplementary Figure 19 consists of two parts: underlying 2D FET modeling and ferroelectric capacitor modeling.

First, we solve the Poisson and drift-diffusion equation for the underlying 2D FET with the same dimensions as those of NC-FET. Electrostatic potential in the channel can be obtained as[17, 18]

$$\frac{d^2\phi}{d^2x} - \frac{\phi}{\lambda^2} + \xi = \frac{q(n_{\mathrm{2D}} - N_{\mathrm{d}})}{\varepsilon_{\mathrm{2d}} t_{\mathrm{2d}}} \qquad \text{(Supplementary Equation 1)}$$

This equation is a modified Poisson’s equation specific for 2D FETs. Where,

$$1/\lambda^2 = 1/\lambda_{\mathrm{t}}^2 + 1/\lambda_{\mathrm{b}}^2,\quad \xi = \frac{V_{\mathrm{int}} - V_{\mathrm{fbt}}}{\lambda_{\mathrm{t}}^2} + \frac{V_{bg} - V_{\mathrm{fbb}}}{\lambda_{\mathrm{b}}^2}$$

$$\frac{1}{\lambda_{\mathrm{t}}^2} = \frac{\varepsilon_{\mathrm{BN}}}{\varepsilon_{\mathrm{2D}} t_{\mathrm{BN}} t_{\mathrm{2D}}},\quad \frac{1}{\lambda_{\mathrm{b}}^2} = \frac{\varepsilon_{\mathrm{SiO2}}}{\varepsilon_{\mathrm{2D}} t_{\mathrm{SiO2}} t_{\mathrm{2D}}}$$

Here, $\phi(x)$ is the electrostatic potential along the channel, q is the elementary charge and N_{d} is the doping concentration in the 2D channel, $\varepsilon_{\mathrm{2D}}/\varepsilon_{\mathrm{BN}}/\varepsilon_{\mathrm{SiO_2}}$ are the dielectric permittivity of 2D semiconductor/top-gate insulator BN/back-gate oxide SiO_2 and $t_{\mathrm{2D}}/t_{\mathrm{BN}}/t_{\mathrm{SiO_2}}$ are the thickness of 2D semiconductor/BN/SiO_2, V_{fbt} and V_{fbb} are the flat-band voltage of top- and back-gate, respectively. For the simulation of CIPS/MoS_2 vdW NC-FETs, t_{BN} is set to zero and an interfacial capacitance induced by the air gap between MoS_2 and CIPS (<1 Å) is included[19]. The channel electron density n_{2D} can be written as

$$n_{2\mathrm{D}} \approx N_{\mathrm{DOS}} \cdot \exp(\frac{q(\phi - V_{\mathrm{ch}})}{kT}) \qquad \text{(Supplementary Equation 2)}$$

where

$$N_{\mathrm{DOS}} = \frac{m^* kT}{2\pi\hbar^2}$$

which is the effective density of states (DOS) for 2D semiconductor, and m^* is the effective electron mass. k is the Boltzmann constant, T is the temperature, and $\hbar$ is the reduced Planck constant. V_{ch} is the channel Fermi potential with the boundary conditions: 0 V at the source and V_{ds} at the drain. According to the gradual channel approximation for the long-channel condition[17], Supplementary Equation 1 can be reduced to

$$n_{2\mathrm{D}} = \frac{\varepsilon_{2\mathrm{D}} t_{2\mathrm{D}}}{q}\left(\xi - \frac{\phi}{\lambda^2}\right) + N_{\mathrm{d}} \qquad \text{(Supplementary Equation 3)}$$

Substituting Supplementary Equation 2 into Equation 3, the electrostatic potential can be expressed as

$$\phi = V_{\mathrm{ch}} + \frac{kT}{q}\ln(\frac{\varepsilon_{2\mathrm{D}} t_{2\mathrm{D}}}{q N_{\mathrm{DOS}}}\left(\xi - \frac{\phi}{\lambda^2}\right) + \frac{N_{\mathrm{d}}}{N_{\mathrm{DOS}}}) \qquad \text{(Supplementary Equation 4)}$$

Supplementary Equation 4 can be solved using the Lambert function method as

$$\phi = \frac{b}{a} + \frac{kT}{q} W_0(\frac{q}{akT} \cdot \exp\left(\frac{q(b - V_{\mathrm{ch}})}{akT}\right)) \qquad \text{(Supplementary Equation 5)}$$

where

$$a = \frac{\varepsilon_{2\mathrm{D}} t_{2\mathrm{D}}}{q N_{\mathrm{DOS}} \lambda^2},\ b = (\frac{\varepsilon_{2\mathrm{D}} t_{2\mathrm{D}} \xi}{q} + N_{\mathrm{d}})/N_{\mathrm{DOS}}$$

Carrier transport is governed by drift-diffusion equation and can be expressed as[17]

$$I_{\mathrm{ds}}(x) = qW n_{2\mathrm{D}}(x)\mu(x)\frac{dV_{\mathrm{ch}}(x)}{dx}$$

where μ is electron mobility, and W is device width. Since current is constant along the channel, it is valid to convert Supplementary Equation 4 into an integral form. Changing variable from V_{ch} to ϕ, we obtain

$$I_{\mathrm{ds}} = q\mu \frac{W}{L}\left[\left(\frac{\varepsilon_{\mathrm{2D}} t_{\mathrm{2D}}}{q}\left(\xi + \frac{kT}{q\lambda^2}\right) + N_{\mathrm{d}}\right)\phi - \frac{\varepsilon_{\mathrm{2D}} t_{\mathrm{2D}}}{q\lambda^2}\frac{\phi^2}{2}\right]_{\phi_{\mathrm{s}}}^{\phi_{\mathrm{d}}}$$

where, L is the channel length and ϕ_{s} (ϕ_{d}) is the electrostatic potential at source (drain) and can be calculated from Supplementary Equation 5 by setting V_{ch}=0 (V_{ch}=V_{ds}).

The overall internal gate charge density (Q_{G}) is contributed by three parts: channel charge (Q_{ch}), parasitic charge due to gate to source/drain coupling (Q_{PS}/Q_{PD}) and back-gate charge (Q_{bg}), which can be expressed as

$$Q_{\mathrm{G}} = Q_{\mathrm{ch}} + Q_{\mathrm{PS}} + Q_{\mathrm{PD}} + Q_{\mathrm{bg}}$$

and $Q_{\mathrm{PS}} = C_{\mathrm{PS}} V_{\mathrm{int}},\ Q_{\mathrm{PD}} = C_{\mathrm{PD}}(V_{\mathrm{int}} - V_{\mathrm{ds}}),\ Q_{\mathrm{bg}} = C_{\mathrm{SiO_2}}\left(V_{\mathrm{bg}} - V_{\mathrm{fbb}} - \frac{\phi_{\mathrm{s}}+\phi_{\mathrm{d}}}{2}\right)$

Channel charge density (Q_{ch}) of the underlying 2D FET is given as[18]

$$Q_{\mathrm{ch}} = qN_{\mathrm{DOS}} \frac{ad\left(\phi_{\mathrm{s}}^2+\phi_{\mathrm{s}}\phi_{\mathrm{d}}+\phi_{\mathrm{d}}^2\right)/3-(b_1 d+ac)(\phi_{\mathrm{s}}+\phi_{\mathrm{d}})/2+b_1 c)}{(c-d(\phi_{\mathrm{s}}+\phi_{\mathrm{d}})/2)}$$

and $b_1 = \frac{\varepsilon_{\mathrm{2D}} t_{\mathrm{2D}} \xi}{qN_{\mathrm{2D}}},\ c = N_d + \frac{\varepsilon_{\mathrm{2D}} t_{\mathrm{2D}}}{q}\left(\xi + \frac{kT}{q\lambda^2}\right),\ d = \frac{\varepsilon_{\mathrm{2D}} t_{\mathrm{2D}}}{q\lambda^2}.$

The electric field in ferroelectric CIPS is related to electric polarization P according to the steady-state Landau–Khalatnikov (L–K) theory[20]

$$E = 2\alpha P + 4\beta P^3 + 6\gamma P^5$$

α, β and γ are Landau coefficients of CIPS, which can be extracted from the P-E curve to be -3.044×10^{8} m/F, 1.035×10^{11} $m^5/F/C^2$ and 8.754×10^{12} $m^9/F/C^4$, respectively, as shown in Supplementary Figure 20a. The above equation can be further written as

$$V_{\mathrm{tg}} - V_{\mathrm{int}} = V_{\mathrm{CIPS}} = t_{\mathrm{CIPS}}(2\alpha P + 4\beta P^3 + 6\gamma P^5) \quad \text{(Supplementary Equation 6)}$$

where t_{CIPS} is the thickness of CIPS. P can be expressed as $P = Q_{\mathrm{G}} - \varepsilon_0 E \approx Q_{\mathrm{G}}$ because $\varepsilon_0 E$ is much smaller than Q_{G}[21], then Supplementary Equation 6 is

approximated as

$V_{\mathrm{int}} = V_{\mathrm{tg}} - t_{\mathrm{CIPS}}(2\alpha Q_{\mathrm{G}} + 4\beta Q_{\mathrm{G}}^3 + 6\gamma Q_{\mathrm{G}}^5)$ (Supplementary Equation 7)

For the calculation of drain current at a particular top-gate voltage in vdW NC-FETs, $I_{\mathrm{ds}}^{\mathrm{NC}}(V_{\mathrm{tg}}^{\mathrm{NC}})$, we follow the method proposed by Junbeom Seo *et al.*[20] and look up the drain current in the underlying 2D FET which was calculated in the previous step, $I_{\mathrm{ds}}^{\mathrm{2D}}(V_{\mathrm{tg}})$, as follows:

$$I_{\mathrm{ds}}^{\mathrm{NC}}\left(V_{\mathrm{tg}}^{\mathrm{NC}}\right) = I_{\mathrm{ds}}^{\mathrm{2D}}(V_{\mathrm{tg}})$$

where the corresponding V_{tg} for underlying 2D FET is given by Supplementary Equation 7 as $V_{\mathrm{tg}} = V_{\mathrm{int}}\left(V_{\mathrm{tg}}^{\mathrm{NC}}\right)$.

Model discussion. Supplementary Figure 20b presents the simulation results based on the model provided above and the extracted Landau coefficients, which exactly match with our experimental results of a vdW NC-FET with the MoS_2 thickness of 4.3 nm, BN thickness of 7.5 nm and CIPS thickness of 48 nm. Supplementary Figure 20c shows the improvement in SS for the vdW NC-FET in comparison with the underlying 2D FET due to the internal gate voltage amplification enabled by the ferroelectric CIPS, as shown in Supplementary Figure 20d.

Supplementary Figure 21 shows the simulated transfer characteristics of vdW NC-FETs with various thickness of BN and CIPS layer. The hysteresis can be reduced by increase t_{BN}. However, it may result in the SS degradation, as shown in Supplementary Figure 21a and b. This is because C_{int} ($C_{\mathrm{int}} = C_{\mathrm{PS}} + C_{\mathrm{PD}} + C_{\mathrm{BN}}C_{\mathrm{2D}}/(C_{\mathrm{BN}} + C_{\mathrm{2D}})$) decrease and the non-hysteresis condition

($C_{\text{int}}<|C_{\text{CIPS}}|$)[22] is improved. The SS degradation can be explained by the reduced internal gate voltage amplification A_{V} ($A_{\text{V}} = \frac{\partial V_{\text{int}}}{\partial V_{\text{tg}}} = \frac{|C_{\text{CIPS}}|}{|C_{\text{CIPS}}| - C_{\text{int}}}$) for vdW NC-FET with a thicker BN layer. The increase of CIPS thickness will decrease $|C_{\text{CIPS}}|$ and leads to an enlarged hysteresis and improved SS, as shown in Supplementary Figure 21c and d.

Supplementary Figure 22a shows the transfer characteristics of a vdW NC-FET at different V_{ds} and the increase of threshold voltage with increasing V_{ds} is observed due to the DIBR effect. The DIBR effect will reduces the I_{ds} in the subthreshold region and cause a characteristic negative differential resistance in the output characteristics, as shown in Supplementary Figure 22b. This can be understood by that an increased drain voltage reduces the total gate charge (Q_{G}) or polarization (P) in CIPS due to the drain to gate coupling, resulting in a decrease in V_{int} and DIBR[13].

The impact of back-gate biasing effect is also examined theoretically, as shown in Supplementary Figure 23. Hysteresis can be suppressed by applying a positive V_{bg} and SS can be improved by a negative V_{bg}, which match with our experimental results. From the equivalent capacitor network of the NC-FET shown in Supplementary Figure 18b, applying a positive V_{bg} will increase the interfacial gate charge, resulting in an increased C_{CIPS}. The increase of C_{CIPS} will reduce the hysteresis but degrade the SS.

Supplementary Note 5

Noise margins of vdW NC-FET inverter

Noise margins of vdW NC-FET inverter were evaluated by extracting V_{IL} (input low), V_{IH} (input high), V_{OL} (output low), and V_{OH} (output high). V_{IL} and V_{IH} are taken from the input voltages, where the slope of the voltage transfer curve is -1 (unity gain), as shown in Supplementary Figure 25. V_{OL} and V_{OH} are extracted using the bi-stable inverter pair logic state definition. The blue curve in Supplementary Figure 25 represents the mirror reflection of transfer curve and the intersection points of these two curves represent two stable states of cross-couple inverters, where V_{OH} and V_{OL} are the corresponding output voltages of these two points. NM_H (high noise margin) and NM_L (low noise margin) are then calculated according to the expressions $NM_H = V_{OH} - V_{IH}$ and $N_{ML} = V_{IL} - V_{OL}$, to be $NM_H = 0.493V_{DD}$ and $NM_L = 0.406V_{DD}$ for $V_{DD} = 1.5$ V.

Supplementary Note 6

CIPS/MoS_2 vdW NC-FETs on polyester substrate

Bending radius determination of flexible NC-FET. The curvature radius of flexible NC-FET can be exactly determined from the optical image of the strained substrate. Supplementary Figure 28 presents the bending radius determination process of a flexible NC-FET in a tensile bending state. Side-view optical image of the bended substrate was taken (Supplementary Figure 28a) and image contour was extracted to locate the position of the substrate (Supplementary Figure 28b). The surface height profile was obtained by calculating the actual distance between the substrate and

bottom edge of the image, shown in Supplementary Figure 28c. A polynomial fit of the extracted height data is finally used to determine the curvature radius at each point via $R_b = \left(1 + \left(\frac{\partial y}{\partial x}\right)^2\right)^{\frac{3}{2}} / \left| \frac{\partial^2 y}{\partial^2 x} \right|$.

Effect of bending radius on top-gate leakage current of flexible NC-FET. Tog-gate leakage current was recorded simultaneously with flexible NC-FETs under various bending curvature radius. As shown in Supplementary Figure 29, I_{tg} increases as the curvature radius decreases. Such a piezotronic effect has been previously reported in vdW ferroelectric In_2Se_3 flake[23], where the Schottky barrier between metal and ferroelectric is modulated by the bound charges induced by the tensile strain. As a major component of the off-state source-drain current, top-gate leakage current increasing leads to the increase of off-state source-drain current for NC-FET with tensile stresses.

Effect of bending cycles on transfer characteristics of flexible NC-FET. To test the bending tolerance of our vdW NC-FET, a bending cycle test was carried out with another device. Flexible substrate was manually bended to ~ 5 mm (not exactly the same at each time) with fingers and then the I_{ds}-V_{tg} characteristics were measured after the substrate was brought back to flat. Supplementary Figure 30a demonstrates the effect of repetitive bending on the performance of vdW NC-FET. It endures repetitive bending up to 500 cycles and no obvious deterioration was observed in I_{ds}-V_{tg} characteristics. The steep switching characteristic with the minimum SS less

than 60 mV/dec was preserved and the hysteresis remained the same level during the cyclic bending test, as shown in Supplementary Figure 30b.

Flexible vdW NC-FETs with different thickness of CIPS. VdW NC-FETs with various thickness of CIPS were fabricated on polyester substrate. Supplementary Figure 26 shows the electrical measurements of two flexible NC-FET with the CIPS thickness of 57 and 35 nm. NC-FET with thinner CIPS exhibits a smaller hysteresis but larger SS, similar to the results of devices on SiO_2/Si substrate. More than 10 vdW NC-FETs on polyester substrate have been fabricated with CIPS thickness from 35 to 89 nm. CIPS thickness dependence of SS and hysteresis are summarized in Supplementary Figure 27. Most devices (8 devices) exhibit SS<60 mV/dec at room temperature.

Supplementary References

1. Si, M. W. et al. Steep-slope hysteresis-free negative capacitance MoS_2 transistors. *Nat. Nanotech.* **13**, 24-28 (2018).
2. Si, M. W. et al. Steep-Slope WSe_2 Negative Capacitance Field-Effect Transistor. *Nano. Lett.* **18**, 3682-3687 (2018).
3. McGuire, F. A., Cheng, Z. H., Price, K. & Franklin, A. D. Sub-60 mV/decade switching in 2D negative capacitance field-effect transistors with integrated ferroelectric polymer. *Appl. Phys. Lett.* **109**, 093101 (2016).
4. Wang, X. D. et al. Two-dimensional negative capacitance transistor with polyvinylidene fluoride-based ferroelectric polymer gating. *npj 2D Mater. Appl.* **1**, 38 (2017).
5. C. Qiu et al., Dirac-source field-effect transistors as energy-efficient, high-performance electronic switches. Science **361**, 387-392 (2018).
6. Castellanos-Gomez, A. et al. Deterministic transfer of two-dimensional materials by all-dry viscoelastic stamping. *2D Mater.* **1**, 011002 (2014).
7. Lee, C. et al. Anomalous Lattice Vibrations of Single- and Few-Layer MoS_2. *Acs Nano* **4**, 2695-2700 (2010).
8. Grzechnik, A., Cajipe, V. B., Payen, C. & McMillan, P. F. Pressure-induced phase transition in ferrielectric $CuInP_2S_6$. *Solid State Commun.* **108**, 43-47 (1998).
9. Ievlev, A. V., Susner, M. A., McGuire, M. A., Maksymovych, P. & Kalinin, S. V. Quantitative Analysis of the Local Phase Transitions Induced by Laser Heating. *Acs Nano* **9**, 12442-12450 (2015).

10. Vysochanskii, Y. M., Stephanovich, V. A., Molnar, A. A., Cajipe, V. B. & Bourdon, X. Raman spectroscopy study of the ferrielectric-paraelectric transition in layered $CuInP_2S_6$. *Phys. Rev. B* **58**, 9119-9124 (1998).
11. Kalinin, S. V., Jesse, S., Tselev, A., Baddorf, A. P. & Balke, N. The Role of Electrochemical Phenomena in Scanning Probe Microscopy of Ferroelectric Thin Films. *Acs Nano* **5**, 5683-5691 (2011).
12. Bark, C. W. et al. Switchable Induced Polarization in $LaAlO_3/SrTiO_3$ Heterostructures. *Nano Lett.* **12**, 1765-1771 (2012).
13. Pahwa, G., Dutta, T., Agarwal, A. & Chauhan, Y. S. Designing energy efficient and hysteresis free negative capacitance FinFET with negative DIBL and 3.5X I_{ON} using compact modeling approach. *Proc. IEEE Eur. Solid-State Device Res. Conf.* 49-54 (2016).
14. Rodriguez, S. et al. A Comprehensive Graphene FET Model for Circuit Design. *IEEE Trans. Electron. Dev.* **61**, 1199-1206 (2014).
15. Das, S. & Appenzeller, J. WSe_2 field effect transistors with enhanced ambipolar characteristics. *Appl. Phys. Lett.* **103**, 103501 (2013).
16. Yusuf, M. H., Nielsen, B., Dawber, M. & Du, X. Extrinsic and Intrinsic Charge Trapping at the Graphene/Ferroelectric Interface. *Nano Lett.* **14**, 5437-5444 (2014).
17. Cao, W., Kang, J. H., Liu, W. & Banerjee, K. A Compact Current-Voltage Model for 2D Semiconductor Based Field-Effect Transistors Considering Interface Traps, Mobility Degradation, and Inefficient Doping Effect. *IEEE Trans. Electron. Dev.*

61, 4282-4290 (2014).

18. Jiang, C. S. et al. A Closed Form Analytical Model of Back-Gated 2-D Semiconductor Negative Capacitance Field Effect Transistors. *IEEE J. Electron. Dev.* **6**, 189-194 (2018).
19. Rooney, A. P. et al. Observing Imperfection in Atomic Interfaces for van der Waals Heterostructures. *Nano Lett*. **17**, 5222-5228 (2017).
20. Landau, L. D. & Khalatnikov, I. M. On the anomalous absorption of sound near a second order phase transition point. *Dokl. Akad. Nauk SSSR.* **96**, 469–472 (1954).
21. Seo, J., Lee, J. & Shin, M. Analysis of Drain-Induced Barrier Rising in Short-Channel Negative-Capacitance FETs and Its Applications. *IEEE Trans. Electron. Dev.* **64**, 1793-1798 (2017).
22. Yeung, C. W., Khan, A. I., Salahuddin, S. & Hu, C. M. Device Design Considerations for Ultra-Thin Body Non-Hysteretic Negative Capacitance FETs. *Proc. Third Berkeley Symp. Energy Efficient Electron. Syst. (E3S)*, 1-2 (2013).
23. Zhou, Y. et al. Out-of-Plane Piezoelectricity and Ferroelectricity in Layered alpha-In_2Se_3 Nanoflakes. *Nano Lett.* **17**, 5508-5513 (2017).
